# Fermatean fuzzy score function and distance measure based group decision making framework for household waste recycling plant location selection

**DOI:** 10.1038/s41598-024-78158-z

**Published:** 2024-11-15

**Authors:** Arunodaya Raj Mishra, Pratibha Rani, Parvaneh Saeidi, Muhammet Deveci, Adel Fahad Alrasheedi

**Affiliations:** 1 Department of Mathematics, Government College Raigaon, Satna, Madhya Pradesh 485441 India; 2 Department of Engineering Mathematics, Koneru Lakshmaiah Education Foundation, Vaddeswaram, Andhra Pradesh India; 3 Research Center in Business, Society, and Technology, ESTec, Faculty of Economic Administrative and Business Sciences, Universidad Tecnológica Indoamérica, Quito, Ecuador; 4 Department of Industrial Engineering, Turkish Naval Academy, National Defence University, 34942 Tuzla, Istanbul Turkey; 5 Royal School of Mines, Imperial College London, London, SW7 2AZ UK; 6 Department of Information Technologies, Western Caspian University, Baku, 1001 Azerbaijan; 7 Statistics and Operations Research Department, College of Science, King Saud University, Riyadh, 11451 Saudi Arabia

**Keywords:** Fermatean fuzzy sets, Distance measure, Household waste recycling plant, MARCOS, Score function, Decision-making, Environmental sciences, Mathematics and computing

## Abstract

The household waste (HW) disposal and recycling have become a significant challenge due to increasing quantities of generated household wastes and increased levels of urbanization. Selecting locations/sites for building new HW recycling plant comprises numerous sustainability dimensions, thus, this work aims to develop new decision-making model for evaluating and prioritizing the HW recycling plant locations. This paper is categorized into three phases. First, we propose new improved score function to compare the Fermatean fuzzy numbers. Moreover, an example is presented to validate the effectiveness of proposed score function over the extant ones. Second, we introduce new distance measure to estimate the discrimination degree between Fermatean fuzzy sets (FFSs) and further discuss its advantages over the prior developed Fermatean fuzzy distance measures. Third, we introduce an integrated methodology by combining the method with the removal effects of criteria (MEREC), the stepwise weight assessment ratio analysis (SWARA) and the measurement alternatives and the ranking according to compromise solution (MARCOS) approaches with Fermatean fuzzy (FF) information, and named as the “FF-MEREC-SWARA-MARCOS” framework. In this method, the FF-distance measure is used to find the weights of involved decision-making experts. Moreover, an integrated criteria weighting method is presented with the combination of MEREC and SWARA models under the context of FFSs, while the combined FF-MEREC-SWARA-MARCOS model is applied to evaluate and prioritize the locations for HW recycling plant development, which illustrates its feasibility of the developed framework. Comparative study and sensitivity assessment are conducted to validate the obtained outcomes. This work provides a hybrid decision analysis approach, which marks a significant impact to the HW recycling plant location selection process with uncertain information.

**Supplementary Information:**

The online version contains supplementary material available at 10.1038/s41598-024-78158-z.

## Introduction

With the rapid urbanization and population growth, annual “household waste (HW)” generation is rapidly increasing in all over the world. The classification of HW activities includes the storage, collection, segregation, transport, disposal and recycling of HW according to the rules or standards^1^. For HW disposal, it is required to segregate them into different categories such as organic, non-hazardous, toxic, e-waste etc. Municipal workers collect waste from different dumping centres into vehicles and send them to recycling plants. The strategic plan of waste management targets the segregation, collection, transportation, disposal, recycle, reuse and recovery of HW in order to reduce the public health and environmental risks^2,3^. Mismanagement of HW can expand the risk of environmental problems such water scarcity, air pollution, soil contamination^4^.

On account of long progression of technological development and significant demand for daily HW disposal, there is a need for constructing new “household waste recycling plants (HWRPs)”. In order to construct a new HWRP, selecting a suitable location is a major concern for the government and non-government organizations^5^. Selection of the most suitable location for HWRP would not only reduce the harmful impacts of global warming but also improve the socio-economic growth of a country. Determination of the HWRPL is a strategic decision, which affects various criteria namely transportation costs, construction costs, job creation, sustainability, customer willingness and awareness and others^6–8^. Therefore, location selection for HWRP development is a “multi-criteria decision making (MCDM)” problem. Due to profound implications of MCDM tools, it can be implemented to address the HWRP locations assessment problem.

In general, data in MCDM problems are imprecise and uncertain because of the subjectivity of human mind and vagueness of available information^9–12^. Zadeh^13^ initiated the “fuzzy set (FS)” doctrine to tackle the uncertain situation, which has been widely utilized for dealing with realistic MCDM problems. Further, several extensions of FS have been developed from various perspectives and implemented in numerous disciplines of real-life problems^14–19^. As a generalization of FS, an idea of “Fermatean fuzzy set (FFS)”^20^ is a novel tool to express the higher levels of uncertainty. In the FFS theory, the cubes sum of belongingness degree (BD) and non-belongingness degree (NBD) of an object is $\le$ 1; therefore, the FFS is more flexible than the FS, the intuitionistic FS (IFS) and the Pythagorean FS (PFS). In comparison with the FSs, IFSs and the PFSs, the FFSs can better describe the uncertainty of complex uncertain problems. Yang et al.^21^ proposed a TOPSIS method with weighted distance measure and presented its utility in green low-carbon port evaluation problem from sustainability and uncertainty perspectives. Mishra et al.^22,23^ firstly analysed the shortcomings of extent works on FFSs. In addition, they combined two different methods for developing a new decision-making framework from Fermatean fuzzy (FF) information viewpoint. With the use of GIS and TOPSIS methods, Hooshangi et al.^24^ evaluated and prioritized the sites for solar farm development in the context of FF information. Zhong et al.^25^ proposed novel Muirhead mean operators for aggregating the FF numbers (FFNs). Based on these operators, they proposed an improved Failure mode and effects analysis approach to detect the possible faults in the production management. Gao et al.^26^ integrated the best worst method (BWM) and VIKOR approach with FFSs to construct a hybrid decision-making methodology for solving healthcare waste treatment method assessment. Golui et al.^27^ modified the Gul et al.’s FF-TOPSIS method^28^ using correlation coefficient in place of distance measure. In this regard, they proposed a new correlation coefficient and presented its properties. Apart from these studies, many theories and applications have been presented using FFSs^29–31^.

In MCDM, criterion weight-finding approaches are characterized as “*Objective and Subjective*” weighting procedures. Numerous authors^32–34^ have presented different objective and subjective weighting procedures. A “method with the removal effects of criteria (MEREC)” is an objective weighting model that utilizes the removal effect of each attribute on the assessment of options and determines the weight of criterion^35,36^. In the literature, several studies have been presented regarding the applications of MEREC method. For example, Haq et al.^37^ suggested a new model by incorporating the single-valued neutrosophic MEREC and MARCOS methods with an application in solving the sustainable material assessment problem. A collective decision model has been put forward with the integration of MEREC, ranking sum and DNMA methods with IFSs. Further, they used their model for evaluating the alternative fuel vehicles selection within the context of IFSs^38^. Ecer and Aycin^39^ recommended an innovative MEREC-based method to measure the innovation performances of G7 countries. Yu et al.^36^ designed new MCDM using the MEREC, the BWM and proximity indexed rating model to evaluate and prioritize offshore wind farm sites from interval 2-tuple linguistic perspective. Yet, no one has incorporated the MEREC, SWARA and MARCOS methods with FFSs for assessing the locations of HWRP development.

For subjective weighting model, Keršuliene et al.^40^ pioneered the idea of “stepwise weight assessment ratio analysis (SWARA)” that has lower computational complexity compared to some other methods. During the process of criteria weight computation, the SWARA model estimates the decision-making experts’ (DMEs’) view related to the importance ratings of criteria. Salamai^41^ incorporated the SWARA and the VIKOR models for the purpose of ranking risks of green supply chain with neutrosophic information. Ayyildiz^42^ developed an innovative Fermatean fuzzy SWARA model for prioritizing the indicators to attain the goals of sustainable development. Further, Stevic^43^ applied the fuzzy SWARA for the assessment of mutual significance of criteria. In addition, they drawn some negative conclusions based on an objective criticism using the fuzzy SWARA model. Pandey and Khurana^44^ studied a hybrid PFSs-based SWARA-COPRAS model for mitigating the risks of industry 4.0. Since its appearance, several researches have been presented regarding the SWARA method^45,46^.

As an innovative and effective approach, the “measurement of alternatives and ranking according to compromise solution (MARCOS)” model measures and prioritizes the options using the compromise solution^47^. In the recent past, several MCDM methodologies have been introduced using MARCOS method. For instance, Torkayesh et al.^48^ presented the hybrid BWM-GIS-MARCOS tool to assess and prioritize the landfill sites for the healthcare waste treatment under the context of grey interval set. Ali^49^ discussed the MARCOS model on q-ROFS settings to deal with the MCDM problem. In 2022, Badi et al.^50^ discussed MARCOS approach to evaluate and prioritize the wind farm site alternatives in relation to different aspects of sustainability. Du et al.^51^ presented an integrated MCDM tool by combining the BWM and the MARCOS models and presented its utility in the assessment of regional distribution network outage loss with reference to numerous indicators. With the use of PFSs, Mishra et al.^22,23^ proposed a decision-making tool by incorporating the objective-subjective weighting model with the MARCOS method and applied for the assessment of sustainable circular suppliers by means of twenty-five criteria. Further, many scholars have extended the conventional MARCOS method on diverse fuzzy sets^52–54^. However, there is no work about the assessment of desirable HWRP locations using a hybrid Fermatean fuzzy decision-making method.

Owing to the broader range of fuzzy information and flexibility in dealing with the practical decision-making problems, this work aims to develop a hybrid MCDM model using FF information and implements to assess the sites for HWRP construction. At present, there is no work which considers the integration of the MEREC, the SWARA and the MARCOS models with FF information for solving HWRPLs problem. Consequently, this study introduces an incorporated decision support system by combining the score function, the distance measure-based model, the MEREC model, the SWARA model and the MARCOS approach with FFSs. Further, we implement the developed methodology for assessing the HWRP locations with respect to different criteria. Based on the abovementioned discussions, the main outcomes of this study are given as.

- To rank the FFNs, new score function is developed, which evades the shortcomings of extant FF-score functions^20,55,56^.
- To quantify the distance between FFSs, new FF-distance measure is proposed. The developed FF-distance measures can deal with the shortcomings of extant FF-distance measures^57–59^.
- An integrated weight-determining model is developed with the combination of MEREC and SWARA methods with FFSs.
- A hybrid FF-MEREC-SWARA-MARCOS methodology is proposed for dealing with the HWRPL selection under sustainability perspective.
- The proposed methodology is applied to a case study of HWRPL assessment problem with FF information, which proves its efficacy and rationality.

Remaining study is summarized as Sect. "Literature review" gives the comprehensive review related to HWRPLs assessment. Section "Proposed FF-score function and distance measure on FFSs" splits into three subsections: (i) some basic definitions are presented related to this study, (ii) new FF-score function is developed to avoid the limitations of some extant FF-score functions and (iii) new FF-distance measure is introduced to evade the shortcomings of extant FF-distance measures. Section "Proposed hybrid FF-MEREC-SWARA-MARCOS method" presents a hybrid MCDM methodology by incorporating the MEREC, the SWARA and the MARCOS approaches with FFSs. Section "Results and discussion" applies the developed FF-MEREC-SWARA-MARCOS model to a case study of HWPRL assessment problem. Moreover, obtained findings are certified by the comparative discussion and sensitivity investigation. Section "Conclusions" discusses the conclusions and the need for further study.

## Literature review

This section highlights the previous studies on location selection problem of waste recycling plant. Existing studies made rich developments on the assessment of different types of waste recycling plant using MCDM approaches. For instance, Shi et al.^7^ incorporated the “genetic algorithm (GA)”-based framework for assessing suitable location of construction waste recycling plant opening. In that study, the authors firstly used GA for getting the fundamental concepts for determining the optimum result. Kumar et al.^60^ used a collective tool by combining the BWM and the VIKOR approach to assess and prioritize the recycling plant sites for “waste electrical and electronic equipment (WEEE)”. Their findings reveal that the plant location selection for WEEE recycling assists to maximize the recovery rate of important assets and minimize the harmful effects on the public health and atmosphere. Zhang et al.^61^ assessed the sites for a HW processing plant development from Pythagorean fuzzy perspective. Moreover, they proposed an innovative Pythagorean fuzzy aggregation operators-based decision model to deal with the problem of HW processing plant location in Shanghai, China. Sheriff et al.^62^ identified the most appropriate site for battery recycling plant construction. In that study, they introduced a hybrid MCDM tool to evaluate the construction plant locations for battery recycling plant from sustainability perspective. Roy et al.^63^ gave the GIS-based MCDM tool for assessing municipal solid waste sites. To assess the recycling center sites for plastic waste in the urban region, Torkayesh and Simic^64^ presented a hybridized MCDM framework by integrating the stratified BWM, “combined compromise solution (CoCoSo)” and the WASPAS with considered criteria. Their research findings concluded that the Pendik district is the optimal site by considering the sustainability dimensions. Till now, very few studies have worked on HWRPLs assessment using uncertain information^61^.

Based on the extant studies, we are unable to find that “which one is the most suitable location for a HWRP construction considering the sustainability aspects?” To conquer the question, the given questions should to be solved:

- What are the prime factors/indicators for selecting the most suitable location under uncertain environment?
- Which is the most significant factor for HW recycling plant location assessment problem?
- Which is the most suitable decision-making method for assessing the sustainable locations for a HWRL?

The main objectives are summarized as.

- To determine the main factors for HWRP locations assessment through literature survey and DMEs’ preferences.
- To introduce a weight-determination model for determining the criteria weights.
- To propose a MCDM model for choosing the suitable sites for a HWRP under FFSs environment.

## Proposed FF-score function and distance measure on FFSs

The current part of this study firstly presents the fundamental definitions and then, proposes a hybrid FF-MEREC-SWARA-MARCOS model to deal with MCDM problems.

### Preliminaries

#### Definition 3.1

Mathematically, an FFS *T* on finite universal set Ω = {*e*_1_, *e*_2_, …, *e*_*n*_} is given as^20^

$$T\, = \,\left\{ {\left. {\left\langle {e_{i} ,\,\left( {\hbar_{T} (e_{i} ),\,{ {\lambda}}_{T} (e_{i} )} \right)} \right\rangle } \right|\,e_{i} \, \in \,\Omega } \right\},$$

wherein $$\hbar_{T} ,\,{{\lambda}}_{T} \,:\,\Omega \, \to \,\left[ {0,\,1} \right]$$ denote the BD and NBD of an object $$e_{i} \, \in \,\Omega$$ to *T*, respectively, satisfying $$0\, \le \,\left( {\hbar_{T} \left( {e_{i} } \right)} \right)^{3} \, + \,\left( {{ {\lambda}}_{T} \left( {e_{i} } \right)} \right)^{3} \, \le \,1.$$ The degree of indeterminacy is $$\pi_{T} \left( {e_{i} } \right) = \,\sqrt[3]{{1\, - \,\hbar_{T}^{3} \left( {e_{i} } \right) - \,{ {\lambda}}_{T}^{3} \left( {e_{i} } \right)}},\,$$$$\forall \,e_{i} \, \in \,\Omega.$$ The term $$\left( {\hbar_{T} (e_{i} ),\,{{\lambda}}_{T} (e_{i} )} \right)$$ is defined as “Fermatean fuzzy number (FFN)”, and simply denoted as $$\alpha = \,\left( {\hbar_{\alpha } ,\,{{\lambda}}_{\alpha } } \right)$$ satisfying $$\hbar_{\alpha } ,\,{{\lambda}}_{\alpha } \, \in \,\left[ {0,\,1} \right]$$ and $$0\, \le \,\hbar_{\alpha }^{3} \, + \,{{\lambda}}_{\alpha }^{3} \, \le \,1.$$

#### Definition 3.2

Consider a FFN $\alpha = \,\left( {\hbar_{\alpha } ,\,{{\lambda}}_{\alpha } } \right).$ Then the score and accuracy functions on given FFN are given^20^ as

1 $${\mathbb{S}}_{SY} \left( \alpha \right) = \,\left( {\left( {\hbar_{\alpha } } \right)^{3} - \left( {{{\lambda}}_{\alpha } } \right)^{3} } \right),$$

2 $$H_{SY} \left( \alpha \right) = \left( {\hbar_{\alpha } } \right)^{3} + \left( {{{\lambda}}_{\alpha } } \right)^{3} ,$$

where ${\mathbb{S}}_{SY} \left( \alpha \right) \in \left[ { - 1,\,1} \right]$ and $H_{SY} \left( \alpha \right) \in \left[ {0,1} \right].$ Further, Rani and Mishra^55^ proposed the normalized score function for a FFN $\alpha = \,\left( {\hbar_{\alpha } ,\,{{\lambda}}_{\alpha } } \right),$ given as

3 $${\mathbb{S}}_{RM} \left( \alpha \right) = \,0.5\,\left( {\left( {\hbar_{\alpha } } \right)^{3} - \left( {{{\lambda}}_{\alpha } } \right)^{3} + \,1} \right).$$

Later, Sahoo^56^ presented an improved score function for a FFN $\alpha = \,\left( {\hbar_{\alpha } ,\,{{\lambda}}_{\alpha } } \right),$ given as

4 $${\mathbb{S}}_{S} \left( \alpha \right) = \,0.5\,\left( {\left( {\hbar_{\alpha } } \right)^{2} - \left( {{{\lambda}}_{\alpha } } \right)^{2} + \,1} \right)\,\left| {\hbar_{\alpha } - \,{{\lambda}}_{\alpha } } \right|.$$

#### Definition 3.3

(Senapati and Yager^20^). Let $$\alpha \, = \,\left( {\hbar_{\alpha } ,\,{{\lambda}}_{\alpha } } \right),$$$$\alpha_{1} \, = \,\left( {\hbar_{{\alpha_{1} }} ,\,{{\lambda}}_{{\alpha_{1} }} } \right)$$ and $$\alpha_{2} \, = \,\left( {\hbar_{{\alpha_{2} }} ,\,{{\lambda}}_{{\alpha_{2} }} } \right)$$ be any three FFNs. Then, some operational laws on given FFNs are presented as

(i) $$\alpha^{c} \, = \,\left( {{{\lambda}}_{\alpha } ,\,\hbar_{\alpha } } \right),$$
(ii) $$\alpha_{1} \, \cap \,\alpha_{2} \, = \,\left( {\left\{ {\hbar_{{\alpha_{1} }} \wedge \hbar_{{\alpha_{2} }} } \right\},\,\left\{ {{{\lambda}}_{{\alpha_{1} }} \vee \,{{\lambda}}_{{\alpha_{2} }} } \right\}} \right),$$
(iii) $$\alpha_{1} \, \cup \,\alpha_{2} \, = \,\left( {\left\{ {\hbar_{{\alpha_{1} }} \vee \hbar_{{\alpha_{2} }} } \right\},\,\left\{ {{{\lambda}}_{{\alpha_{1} }} \, \wedge {{\lambda}}_{{\alpha_{2} }} } \right\}} \right),$$
(iv) $$\alpha_{1} \, \oplus \,\alpha_{2} \, = \,\left( {\sqrt[3]{{\hbar_{{\alpha_{1} }}^{3} \, + \,\hbar_{{\alpha_{2} }}^{3} \, - \,\hbar_{{\alpha_{1} }}^{3} \,\hbar_{{\alpha_{2} }}^{3} }},\,\,{{\lambda}}_{{\alpha_{1} }} \,{{\lambda}}_{{\alpha_{2} }} } \right),$$
(v) $$\alpha_{1} \, \otimes \,\alpha_{2} \, = \,\left( {\hbar_{{\alpha_{1} }} \,\hbar_{{\alpha_{2} }} ,\,\sqrt[3]{{{{\lambda}}_{{\alpha_{1} }}^{3} \, + \,{{\lambda}}_{{\alpha_{2} }}^{3} \, - \,{ {\lambda}}_{{\alpha_{1} }}^{3} \,{{\lambda}}_{{\alpha_{2} }}^{3} }}} \right),$$
(vi) $$\gamma \,\alpha \, = \,\left( {\sqrt[3]{{1 - \left( {1 - \,\hbar_{\alpha }^{3} } \right)^{\gamma } }}\,,\,\left( {{{\lambda}}_{\alpha } } \right)^{\gamma } } \right),\,\,\gamma > \,0,$$
(vii) $$\alpha^{\gamma } \, = \,\left( {\left( {\hbar_{\alpha } } \right)^{\gamma } ,\,\sqrt[3]{{1 - \left( {1 - \,{{\lambda}}_{\alpha }^{3} } \right)^{\gamma } }}} \right),\,\,\gamma \, > \,0.$$

### New score function for FFNs

For $p>1$, a new FF-score function is defined for a FFN $\alpha \, = \,\left( {\hbar_{\alpha } ,\,{{\lambda}}_{\alpha } } \right).$

5 $${\mathbb{S}}\left( \alpha \right)\, = \,\left( {\frac{{(\hbar^{3} )^{p} + \,(1 - \,{{\lambda}}^{3} )^{p} }}{2}} \right)^{{{\raise0.7ex\hbox{$1$} \!\mathord{\left/ {\vphantom {1 p}}\right.\kern-0pt} \!\lower0.7ex\hbox{$p$}}}} .$$

The developed FF-score function, given by Eq. (5), holds the following properties:

#### Theorem 3.1

*The developed FF-score function, given by Eq. (*5*), is monotonically increasing over* $\hbar$* and monotonically decreasing over* ${{\lambda}}.$

#### Proof

The proof of this theorem is given in Section S1 of supplementary file.

#### Theorem 3.2

The developed FF-score function of a FFN $\alpha \, = \,\left( {\hbar_{\alpha } ,\,{{\lambda}}_{\alpha } } \right)$ fulfils the following results:

(p1) $$\mathbb{S}\left( {0,\,1} \right) = \,0$$ and $$\mathbb{S}\left( {1,\,0} \right) = \,1.$$

(p2) $$0\,\, \le \mathbb{S} \left( \alpha \right) \le \,1.$$

#### Proof

The proof of this theorem is given in Section S1 of supplementary file.

#### Example 3.1

Let us consider $\alpha_{1} = \,\left( {0.4,\,0.4} \right)$ and $\alpha_{2} = \,\left( {0.5,\,0.5} \right)$ be the given FFNs. It can be noted that the prior developed FF-score functions by Senapati and Yager^20^, Rani and Mishra^55^ and Sahoo^56^ cannot discriminate the given FFNs because ${\mathbb{S}}_{SY} \left( {\alpha_{1} } \right) = {\mathbb{S}}_{SY} \left( {\alpha_{2} } \right) = \,\,0,$ ${\mathbb{S}}_{RM} \left( {\alpha_{1} } \right) = {\mathbb{S}}_{RM} \left( {\alpha_{2} } \right) = \,\,0.5$ and ${\mathbb{S}}_{S} \left( {\alpha_{1} } \right) = {\mathbb{S}}_{S} \left( {\alpha_{2} } \right) = \,\,0.$ However, the proposed FF-score function computes the results as ${\mathbb{S}}\left( {\alpha_{1} } \right) = \,\,0.6634$ and ${\mathbb{S}}\left( {\alpha_{2} } \right)\, = \,0.625.$ Thus, $\alpha_{1} \,\, > \,\alpha_{2} .$ It implies that introduced FF-score function (4) can effectively distinguish the considered FFNs.

### New FF-distance measure

To determine the dissimilarity degree between FFSs, a new FF-distance measure is proposed, which can successfully handle the shortcomings of extant FF-distance measures^57–59^. Let *S* and* T* be two FFSs. Then new FF-distance measure is given by

6 $$\,d\left( {S,\,T} \right)\, = \frac{1}{4t}\,\sum\limits_{i\, = 1}^{t} {\left[ \begin{gathered} \left| {\hbar_{S}^{3} (e_{i} ) - \,\hbar_{T}^{3} (e_{i} )} \right| + \,\left| {\lambda_{S}^{3} (e_{i} ) - \,\lambda_{T}^{3} (e_{i} )} \right| + \left| {\hbar_{S}^{3} (e_{i} )\,\lambda_{T}^{3} (e_{i} )\, - \,\hbar_{T}^{3} (e_{i} )\,\lambda_{S}^{3} (e_{i} )} \right|\, \hfill \\ + \left( {\left| {\min \left\{ {\hbar_{S}^{3} (e_{i} ),\,\,\lambda_{T}^{3} (e_{i} )\,} \right\} - \,\min \left\{ {\hbar_{T}^{3} (e_{i} ),\,\,\lambda_{S}^{3} (e_{i} )} \right\}} \right|} \right. \hfill \\ \left. { + \left| {\max \left\{ {\hbar_{S}^{3} (e_{i} ),\,\,\lambda_{T}^{3} (e_{i} )\,} \right\} - \,\max \left\{ {\hbar_{T}^{3} (e_{i} ),\,\,\lambda_{S}^{3} (e_{i} )} \right\}} \right|} \right) \hfill \\ \end{gathered} \right]} .$$

#### Theorem 3.3

For $S,\,T\, \in \,FFSs\left( \Omega \right),$ the real-valued function $d\left( {S,\,T} \right)$ satisfies the given properties:

 (a_1_). $$0 \le d\left( {S,\,T} \right) \le 1,$$

(a_2_). $$d\left( {S,\,T} \right) = d\left( {T,\,S} \right),$$

(a_3_). $$d\left( {S,\,T} \right) = 0 \Leftrightarrow \,S\, = \,T,$$

(a_4_). If $$R\, \subseteq \,S \subseteq \,T,$$ then $$d\left( {R,\,T} \right) \ge d\left( {R,\,S} \right)$$ and $$d\left( {R,\,T} \right) \ge d\left( {S,\,T} \right),$$$$\forall \,\,R,\,S,\,T\,\, \in FFSs\left( \Omega \right).$$

#### Proof

The proof is given in Section S1 of supplementary file.

In order to verify the efficiency of introduced distance measure $d\left( {S,\,T} \right),$ we compare it with various extant measures as^57–59^. The computational results are discussed in Section S2 of supplementary file. For this purpose, we take some FFSs to execute the experimental results of introduced and extant measures. From Table 1, we can see that the previously developed measures *d*_*A*1_ (*S*, *T*), *d*_*A*2_ (*S*, *T*), *d*_*A*3_ (*S*, *T*), *d*_*A*4_ (*S*, *T*), *d*_*A*5_ (*S*, *T*), *d*_*A*6_ (*S*, *T*) (we take *α* = 0.4, *β* = 0.6), *d*_*A*7_ (*S*, *T*), *d*_*G*1_ (*S*, *T*), *d*_*G*2_ (*S*, *T*), *d*_*G*3_ (*S*, *T*), *d*_*G*4_ (*S*, *T*) and *d*_*k*_ (*S*, *T*) present unreasonable results. In the line, we discuss some drawbacks of previously developed measures *d*_*A*1_ (*S*, *T*), *d*_*A*2_ (*S*, *T*), *d*_*A*3_ (*S*, *T*), *d*_*A*4_ (*S*, *T*), *d*_*A*5_ (*S*, *T*), *d*_*A*6_ (*S*, *T*), *d*_*A*7_ (*S*, *T*), *d*_*G*1_ (*S*, *T*), *d*_*G*2_ (*S*, *T*), *d*_*G*3_ (*S*, *T*), *d*_*G*4_ (*S*, *T*) and *d*_*k*_ (*S*, *T*):

- Ashraf et al.’s measure *d*_*A*6_ (*S*, *T*) presents the division by zero problem for the sets *S* = {(*e*_1_, 1.0, 0.0)} and *T* = {(*e*_1_, 0.0, 0.0)}.
- For the Cases 1 and 5, Ashraf et al.’s measure *d*_*A*2_ (*S*, *T*) does not hold the property (a_3_) of FF-distance measure. Additionally, other measures present the following results: *d*_*A*1_ (*S*, *T*) = *d*_*A*4_ (*S*, *T*) = *d*_*A*7_ (*S*, *T*) = *d*_*G*1_ (*S*, *T*) = *d*_*G*2_ (*S*, *T*) = *d*_*G*3_ (*S*, *T*) = *d*_*G*4_ (*S*, *T*) = 0 (in Case-3) and *d*_*A*6_ (*S*, *T*) = *d*_*A*7_ (*S*, *T*) = 0 (in Case-4), which are indeed not a crisp number.
- Previously developed measures *d*_*A*3_ (*S*, *T*), *d*_*A*4_ (*S*, *T*), *d*_*A*5_ (*S*, *T*), *d*_*A*6_ (*S*, *T*), *d*_*A*7_ (*S*, *T*), *d*_*G*1_ (*S*, *T*), *d*_*G*2_ (*S*, *T*), *d*_*G*3_ (*S*, *T*), *d*_*G*4_ (*S*, *T*) (in Case-1 and Case-2) have no capabilities to define the positive discrimination from negative discrimination. Similar cases occur for *d*_*A*4_ (*S*, *T*) and *d*_*A*5_ (*S*, *T*) (in Case-5 and Case-6).

**Table 1:** Computational results of the proposed and extant distance measures (Bold and highlighted cell shows unreasonable results and “NaN” represents division by zero problems).

*S* *T*	Case-1 {(*e*_1_,0.3,0.3)} {(*e*_1_,0.4,0.4)}	Case-2 {(*e*_1_, 0.3, 0.4)} {(*e*_1_, 0.4, 0.3)}	Case-3 {(*e*_1_, 1, 0)} {(*e*_1_, 0, 0)}	Case-4 {(*e*_1_, 0.5, 0.5)} {(*e*_1_, 0, 0)}	Case-5 {(*e*_1_,0.4,0.2)} {(*e*_1_,0.5,0.3)}	Case-6 {(*e*_1_,0.4,0.2)} {(*e*_1_,0.5,0.2)}
*d* (*S*, *T*)	0.018	0.038	0.5	0.062	0.04	0.031
*dA*1 (*S*, *T*)	0.074	0.037	**1**	0.25	0.08	0.061
*dA*2 (*S*, *T*)	**0**	0.037	0.5	**0**	0.021	0.03
*dA*3 (*S*, *T*)	**0.037**	**0.037**	0.75	0.125	0.05	0.046
*dA*4 (*S*, *T*)	**0.037**	**0.037**	**1**	0.125	**0.061**	**0.061**
*dA*5 (*S*, *T*)	**0.929**	**0.929**	**0**	0.778	**0.885**	**0.885**
*dA*6 (*S*, *T*)	**0.578**	**0.578**	**NaN**	**1**	0.617	0.195
*dA*7 (*S*, *T*)	**0.578**	**0.578**	**1**	**1**	0.526	0.459
*dG*1 (*S*, *T*)	**0.071**	**0.071**	**1**	0.222	0.078	0.061
*dG*2 (*S*, *T*)	**0.073**	**0.073**	**1**	0.234	0.079	0.061
*dG*3 (*S*, *T*)	**0.074**	**0.074**	**1**	0.25	0.08	0.061
*dG*4 (*S*, *T*)	**0.074**	**0.074**	**1**	0.246	0.08	0.061
*dk* (*S*, *T*)	0.315	0.305	0.854	0.389	0.325	0.316

Thus, it follows that the proposed FF-distance measure *d* (*S*, *T*) is more robust than various extant FF-distance measures.

## Proposed hybrid FF-MEREC-SWARA-MARCOS method

**Fig. 1:**
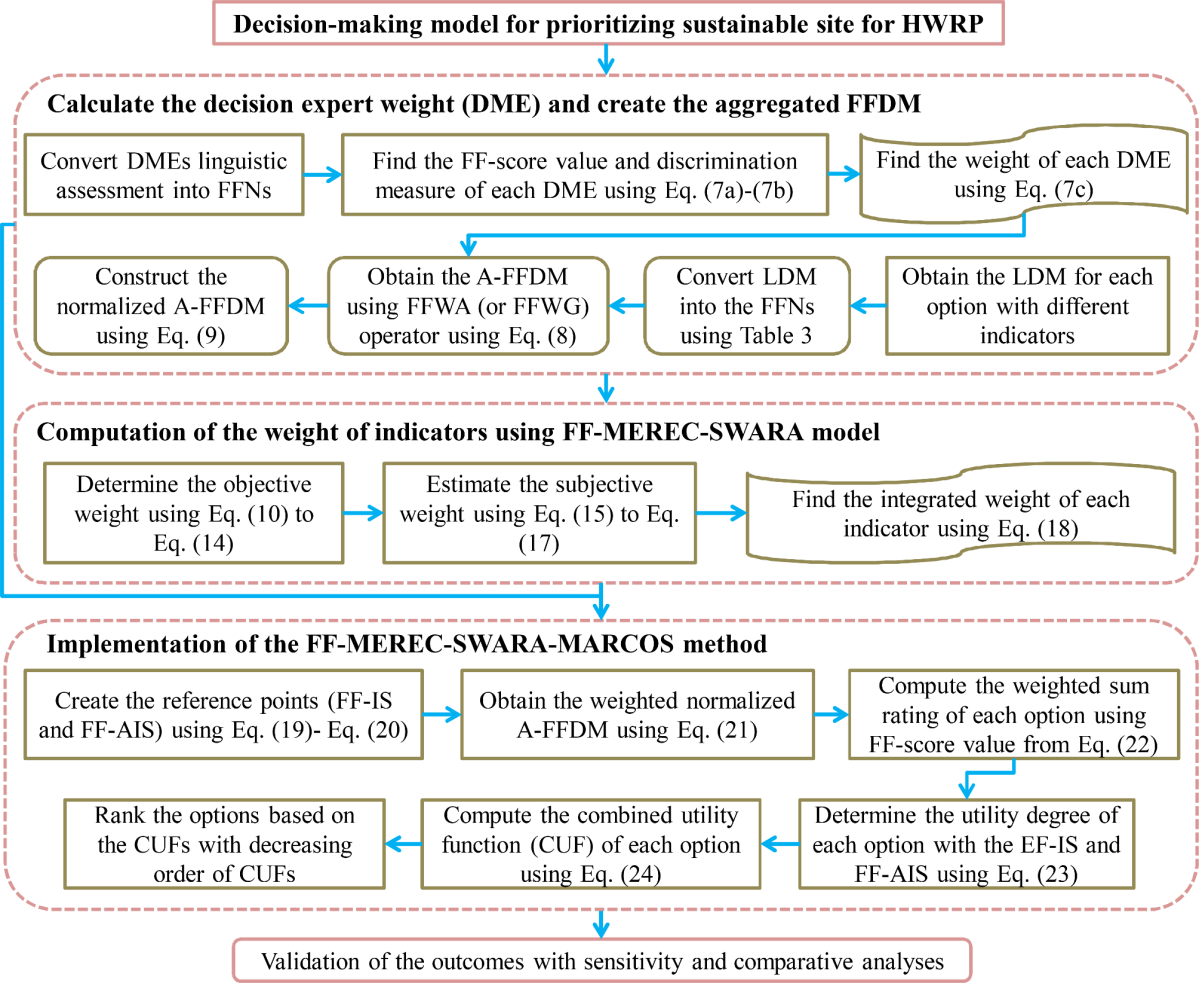
Flowchart of proposed decision-making methodology.

The present section develops a hybrid MARCOS method based on the combination of DMEs’ weighting model, the FF-weighted averaging operator, and an integrated objective-subjective weighting model with FF information. Figure 1 presents the pictorial representation of the proposed framework. This method involves the following steps:

**Step 1:** Formation of “qualitative decision matrix (QDM)”.

A panel $\,\left\{ {g_{1} ,\,g_{2} ,\,...,\,g_{\ell } } \right\}$ of DMEs is formed to assess a set of options $F\, = \,\left\{ {F_{1} ,\,F_{2} ,\,...,\,F_{p} } \right\}$ over the criteria set $V\, = \,\left\{ {V_{1} ,\,V_{2} ,\,...,\,V_{q} } \right\}$. Here, each DME presents his/her views for the performance of each option concerning diverse criteria in the form of “qualitative term (QT)” as Likert scale, which are given in Tables 2, 3 and adopted from Rani and Mishra^55^. Let $\Xi \, = \,\left[ {o_{ij}^{(k)} } \right]_{p \times q}$ be the “qualitative decision-matrix (QDM)”, in which $o_{ij}^{(k)}$ specifies the rating of each option *F*_*i*_ over criterion *V*_*j*_ by *k*th DME.

**Table 2:** Ratings of attributes and DMEs in QTs.

QTs	FFNs
Extremely skilled (ES)	(0.9,0.2)
Much skilled (MS)	(0.8,0.4)
Skilled (S)	(0.7,0.5)
Slight skilled (SS)	(0.6,0.7)
Less skilled (LS)	(0.5,0.8)

**Table 3:** QTs of the alternatives over diverse criteria for HWRPLs selection.

QTs	FFNs
Extremely preferred (EP)	(0.95,0.2)
Most preferred (MP)	(0.9,0.3)
Preferred (P)	(0.85,0.4)
Quite preferred (QP)	(0.8,0.5)
Slightly preferred (SP)	(0.75,0.6)
Average (A)	(0.6,0.7)
Slightly unreferred (SU)	(0.5,0.8)
Quite unreferred (QU)	(0.4,0.85)
Unreferred (U)	(0.3,0.9)
Much unreferred (MU)	(0.2,0.95)

**Step 2:** Estimation of weight of experts.

To find the weight of DME, various techniques have been discussed. Some of them only focus on the score function-based formula or merely pay attention on discrimination information of DMEs. In this study, we present a combined weight model to obtain the weight of DME using the FF-score function-based method and proposed FF-distance measure.

Based on QT and associated FFN $g_{k} = \left( {\hbar_{k} ,{{\lambda}}_{k} } \right)$, we determine the individual assessment degree of each DME using FF-score value and given as

7a $$\Phi_{k}^{1} = \frac{{\hbar_{k}^{3} \left( {2 - \hbar_{k}^{3} - {{\lambda}}_{k}^{3} } \right)}}{{\sum\limits_{k = 1}^{\ell } {\left[ {\hbar_{k}^{3} \left( {2 - \hbar_{k}^{3} - {{\lambda}}_{k}^{3} } \right)} \right]} }},\forall \,\,k = 1,2,...,\ell .$$

Here, $$\Phi_{k}^{1} \ge \,0$$ and $$\sum\limits_{k\, = 1}^{\ell } {\Phi_{k}^{1} \, = \,1.}$$

We compute the discrimination degree of each DME with developed FF-distance measure to estimate the normalized weight of DME as follows:

7b $$\Phi_{k}^{2} = \frac{{\frac{1}{k - 1}\sum\limits_{t = 1}^{\ell } {d\left( {g_{k} ,\,g_{t} } \right)} }}{{\sum\limits_{k = 1}^{\ell } {\left( {\frac{1}{l - 1}\sum\limits_{t = 1}^{\ell } {d\left( {g_{k} ,\,g_{t} } \right)} } \right)} }},\,\,\,k = 1,2,...,\ell .$$

Here, $$\Phi_{k}^{2} \ge \,0$$ and $$\sum\limits_{k\, = 1}^{\ell } {\Phi_{k}^{2} \, = \,1.}$$

Hence, the combined weight $\Phi_{k}$ for each DME can be calculated in the following expression:

7c $$\Phi_{k} = \frac{{\Phi_{k}^{1} *\Phi_{k}^{2} }}{{\sum\nolimits_{k = 1}^{\ell } {\Phi_{k}^{1} *\Phi_{k}^{2} } }},$$

Here, $\Phi_{k} \, \ge \,0$ and $\sum\limits_{k\, = 1}^{\ell } {\Phi_{k} \, = \,1.}$ Thus, the higher the rating of Eq. (7c) is, the larger weight should be given to the DME *g*_*k*_, *k* = 1, 2, …, ${\ell}$.

**Step 3:** Aggregate the individual opinions.

To aggregate the QDM $\Xi \, = \,\left[ {o_{ij}^{(k)} } \right]_{p \times q} ,$ FF-weighted averaging operator^20^ is implemented on QDM and obtained an aggregated FF-decision matrix (A-FF-DM) $A = \left( {\upsilon_{ij} } \right)_{p\, \times \,q}$, where

8 $$\upsilon_{ij} = \left( {\hbar_{ij} ,\,{{\lambda}}_{ij} } \right) = FFWA_{\Phi_{k} } \,\left( {o_{ij}^{(1)} ,\,o_{ij}^{(2)} ,...,o_{ij}^{(\ell )} } \right) = \left( {\sqrt[3]{{1 - \prod\limits_{k = 1}^{\ell } {\left( {1 - \left( {\hbar_{ij}^{\left( k \right)} } \right)^{3} } \right)^{{\Phi_{k} }} \,} }},\,\,\prod\limits_{k = 1}^{\ell } {\left( {{{\lambda}}_{ij}^{\left( k \right)} } \right)^{{\Phi_{k} }} } } \right).$$

**Step 4:** Determination of weight of criteria.

Let $W = \left( {\omega_{1} ,\,\omega_{2} ,...,\,\omega_{q} } \right)^{T}$ be a collection of criteria weights satisfying $\sum\limits_{j = 1}^{q} {\omega_{j} } \, = 1$ and $\,\omega_{j} \in \left[ {0,\,\,1} \right].$ Then, a scheme for finding weight of criteria is discussed as follows:

**Case 1:** Obtain the objective weight of attributes.

**Step 4.1:** Normalization of the A-FF-DM.

If the A-FF-DM contains cost and benefit types of criteria, then it is needed to create the normalized A-FF-DM (NA-FF-DM) ${\mathbb{N}} = \left( {\varsigma_{ij} } \right)_{p\, \times \,q} ,$ where $\varsigma_{ij}$ is a normalized FFN, defined by

9 $$\varsigma_{ij} = \left( {\overline{\hbar }_{ij} ,\overline{{ {\lambda}}}_{ij} } \right) = \left\{ \begin{gathered} \upsilon_{ij} = \left( {\hbar_{ij} ,\,{{\lambda}}_{ij} } \right),\,\,\,\,\,\,\,\,j \in V_{b} , \hfill \\ \left( {\upsilon_{ij} } \right)^{c} = \left( {{{\lambda}}_{ij} ,\hbar_{ij} } \right),\,\,j \in V_{n} , \hfill \\ \end{gathered} \right.$$

where *V*_*b*_ is benefit type criterion and *V*_*n*_ is cost type criterion.

**Step 4.2:** Computation of FF-score matrix.

Applying Eq. (5) to develop the FF-score matrix $\Theta = \left( {\delta_{ij} } \right)_{p\, \times \,q}$ of each FFN $\varsigma_{ij}$ using Eq. (10) as follows:

10 $$\delta_{ij} = \,\left( {\frac{{(\overline{\hbar }_{ij}^{3} )^{p} + \,(1 - \,\overline{{{\lambda}}}_{ij}^{3} )^{p} }}{2}} \right)^{{{\raise0.7ex\hbox{$1$} \!\mathord{\left/ {\vphantom {1 p}}\right.\kern-0pt} \!\lower0.7ex\hbox{$p$}}}} .$$

**Step 4.3:** Define performance of option.

By means of Step 4.2, the performance of each option over considered attributes is revealed in Eq. (11) as

11 $$\Psi_{i} = \,ln\left( {1 + \,\left( {\frac{1}{q}\,\sum\limits_{j} {\left| {ln\left( {\delta_{ij} } \right)} \right|} } \right)} \right).$$

**Step 4.4:** Estimation of performance of option removing of each criterion.

The assessment degree of *i*th option by eliminating *j*th attribute, discussed as

12 $$\Psi_{ij}^{\prime} = ln\left( {1 + \left( {\frac{1}{q}\sum\limits_{k,k \ne j} {\left| {ln\left( {\delta_{ik} } \right)} \right|} } \right)} \right).$$

**Step 4.5:** Computation of absolute deviation of each attribute.

We compute the deviations of *j*th criterion with the Step 4.3 and Step 4.4. Let $\mho_{j}$ denotes the deviation of *j*th criterion, then

13 $$\mho_{j} = \sum\limits_{i} {\left| {\Psi_{ij}^{\prime} - \Psi_{i} } \right|} .$$

**Step 4.6:** Find the weight value of each criterion.

Considering the absolute deviation, we estimate the weight of each attribute as

14 $$\omega_{j}^{o} = \frac{{\mho_{j} }}{{\sum\limits_{j = 1}^{q} {\mho_{j} } }}.$$

**Case 2:** Determination of subjective weight with the FF-SWARA model.

The SWARA method firstly finds the score value of each criterion using the DMEs’ opinions. On the basis of obtained FF-score values, arrange the criteria from higher to lower FF-score values. This process comprises following phases:

**Step 4.7:** Each DME assesses the criteria set and presents their opinions regarding each criterion in terms of LVs. With the help of FFWA operator, find the aggregated value $G = \left( {\alpha_{j} } \right)_{1 \times \,q} ,$ of criteria performances given by each DME as follows:

$$G = \left( {\alpha_{j} } \right)_{1 \times \,q} = FFWA_{\Phi_{k}} \left( {\alpha_{j}^{\left( 1 \right)} ,\alpha_{j}^{\left( 2 \right)} ,...,\alpha_{j}^{\left( \ell \right)} } \right),\,j = 1,2,...,q.$$

Also, we compute the FF-score rating using Eq. (5) for each criterion.

**Step 4.8:** Determine the relative importance (*s*_*j*_) of attribute using FF-score rating.

**Step 4.9:** Find the comparative coefficient using Eq. (15), and given by

15 $$k_{j} = \left\{ \begin{gathered} 1,\,\,\,\,\,\,\,\,\,\,\,\,\,j = 1, \hfill \\ s_{j} + 1,\,\,j > 1. \hfill \\ \end{gathered} \right.$$

**Step 4.10:** Calculate the weight of attribute using the expression (16) as

16 $$r_{j} = \left\{ \begin{gathered} 1,\,\,\,\,\,\,\,\,\,j = 1 \hfill \\ \frac{{r_{j - 1} }}{{k_{j} }},\,\,j > 1 \hfill \\ \end{gathered} \right.$$

**Step 4.11:** Computation of overall weight of attribute with Eq. (17), and given by

17 $$\omega_{j}^{s} = \frac{{r_{j} }}{{\sum\nolimits_{j = 1}^{q} {r_{j} } }},\,j = 1,2,...,q.$$

**Case 3:** Based on Case 1 and Case 2, we compute the aggregated or combined weight given by

18 $$\omega_{j} = \vartheta \,\omega_{j}^{s} + \left( {1 - \vartheta } \right)\omega_{j}^{o} ,$$

where *ϑ* is the aggregating coefficient of decision precision parameter within the range of 0 to 1.

**Step 5:** Express the positive-ideal rating (PIR) and negative-ideal rating (NIR) on FFNs using Eq. (19) and Eq. (20), respectively, as

19 $$\alpha_{j}^{ + } \, = \left( {\hbar_{j}^{ + } ,\,{{\lambda}}_{j}^{ + } } \right) = \,\left\{ \begin{gathered} \left( {\mathop {\max }\limits_{i} \,\hbar_{ij} ,\mathop {\min }\limits_{i} \,{{\lambda}}_{ij} } \right),\,\,\,j\, \in \,V_{b} ,\,\, \hfill \\ \left( {\mathop {\min }\limits_{i} \,\hbar_{ij} \mathop {\max }\limits_{i} \,{{\lambda}}_{ij} } \right),\,\,\,j\, \in \,V_{n} . \hfill \\ \end{gathered} \right.\,\,$$

20 $$\alpha_{j}^{ - } \, = \left( {\hbar_{j}^{ - } ,\,{{\lambda}}_{j}^{ - } } \right) = \,\left\{ \begin{gathered} \left( {\mathop {\min }\limits_{i} \,\hbar_{ij} ,\mathop {\max }\limits_{i} \,{{\lambda}}_{ij} } \right),\,\,\,j\, \in \,V_{b} , \hfill \\ \left( {\mathop {\max }\limits_{i} \,\hbar_{ij} ,\mathop {\min }\limits_{i} \,{{\lambda}}_{ij} } \right),\,\,j\, \in \,V_{n} . \hfill \\ \end{gathered} \right.$$

**Step 6:** Compute the weighted normalized A-FF-DM (WNA-FF-DM).

We obtain the WNA-FF-DM $${\mathbb{N}}_{w} = \left( {\overset{\lower0.5em\hbox{$\smash{\scriptscriptstyle\frown}$}}{\varsigma }_{ij} } \right)_{p\, \times \,q}$$ as

21 $$\overset{\lower0.5em\hbox{$\smash{\scriptscriptstyle\frown}$}}{\varsigma }_{ij} = \left( {\overset{\lower0.5em\hbox{$\smash{\scriptscriptstyle\frown}$}}{\hbar }_{ij} ,\,\overset{\lower0.5em\hbox{$\smash{\scriptscriptstyle\frown}$}}{{{\lambda}}}_{ij} } \right) = \omega_{j} \,\varsigma_{ij} = \,\left( {\sqrt[3]{{1 - \left( {1 - \,\left( {\overline{\hbar }_{ij} } \right)^{3} } \right)^{{\omega_{j} }} }}\,,\,\left( {\overline{{{\lambda}}}_{ij} } \right)^{{\omega_{j} }} } \right).$$

**Step 7:** Estimation of the FF-score rating of the WNA-FF-DM using Eq. (5) as

22 $$\Delta_{i} \, = \,\sum\limits_{j = 1}^{q} {{\mathbb{S}}\left( {\overset{\lower0.5em\hbox{$\smash{\scriptscriptstyle\frown}$}}{\varsigma }_{ij} } \right)} ,\,i = 1,2,...,p,$$

wherein $${\mathbb{S}}\left( {\overset{\lower0.5em\hbox{$\smash{\scriptscriptstyle\frown}$}}{\varsigma }_{ij} } \right)$$ means the proposed FF-score rating using Eq. (5).

**Step 8:** Finding utility degree (UD) of options with the PIR and NIR as

23 $$m_{i}^{ - } \, = \,\frac{{\Delta_{i} }}{{\Delta_{ais} }}\;{\text{and}}\;m_{i}^{ + } \, = \,\frac{{\Delta_{i} }}{{\Delta_{is} }},$$

where $\Delta_{is}$ and $\Delta_{ais}$ denote summation of FF-score ratings of weighted PIR $\alpha_{jw}^{ + }$ and NIR $\alpha_{jw}^{ - } ,$ respectively.

**Step 9:** Assessing combined utility function (CUF).

the CUF of each alternative with weighted PIR and NIR is obtained as.

24 $$g\left( {m_{i} } \right)\, = \,\frac{{m_{i}^{ + } + \,m_{i}^{ - } }}{{1 + \,\frac{{1 - g\left( {m_{i}^{ + } } \right)}}{{g\left( {m_{i}^{ + } } \right)}} + \frac{{1 - g\left( {m_{i}^{ - } } \right)}}{{g\left( {m_{i}^{ - } } \right)}}}},\;{\text{where}}\;g\left( {m_{i}^{ + } } \right) = \,\frac{{m_{i}^{ - } }}{{m_{i}^{ - } + m_{i}^{ + } }}\;{\text{and}}\;g\left( {m_{i}^{ - } } \right) = \,\frac{{m_{i}^{ + } }}{{m_{i}^{ - } + m_{i}^{ + } }},\,i = 1,2,...,p.$$

**Step 10:** Prioritizing options with the CUFs and choosing the suitable one with highest CUF rating.

## Results and discussion

This section first shows a case study of HWRPLs problem and further implements the proposed FF-MEREC-SWARA-MARCOS approach for choosing an appropriate location for HWRP. Next, it presents the comparison and sensitivity analysis to confirm the obtained findings. Lastly, we discuss the implications of the proposed work.

### Case study: household waste recycling plant location (HWRPL) selection

Increasing quantity and complexity of HW concerns about recycling of waste materials. India faces many environmental challenges due to HW mismanagement. In the case study, we have chosen Indore region, cleanest and leading city in the Madhya Pradesh (India) to locate the HWRP. To this aim, we consider a case study of HWRPL selection of an Indian company, located in Indore. This enterprise has been working for last 15 years and is recognized as a market frontrunner in its waste recycling facilities. Due to increasing amount of HW, this enterprise needs to create a new HWRP but it does not have any appropriate system for constructing location. In this work, we concentrate on the application of robust methodology for assessing the HWRPLs which will help the DMEs to assess an appropriate location for HWRP.

**Fig. 2:**
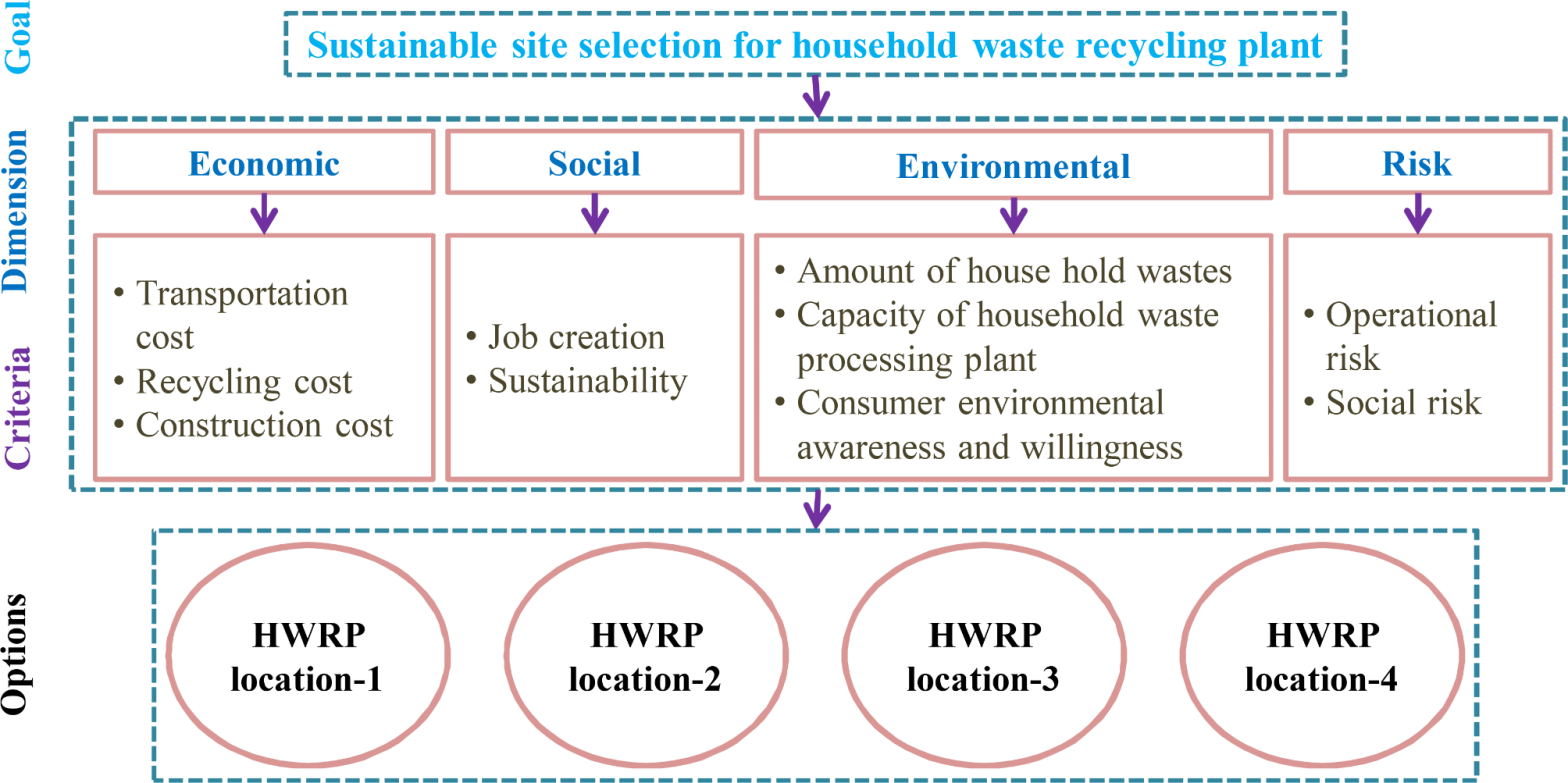
Hierarchical structure of the HWRPL assessment based on 10 indicators/factors.

To collect the data for the assessment and investigation, we planned in-person meetings with the DMEs. Though we invited nine DMEs and out of which, four DMEs are approved to collaborate with us during the preparation of questionnaires. In the committee of four DMEs, each DME has more than 12 years’ expertise in the discipline of MSW management, sustainability and ecological planning and gave their views in taking an appropriate decision. Out of which two of them are from the MSW management, one DME is from sustainability and the other one is from ecological planning. The DMEs supported with scholars during the complete study. They planned some strategies that can be executed in other HWRPL selection problem in India. Then, we have studied the related literature to choose the HWRPLs as alternatives in India. Lastly, we have determined 10 criteria, which are denoted as *V*_1_, *V*_2_, …, *V*_10_. In this study, economic, social, environmental and risk aspects are considered for assessing the HWRPLs. After preliminary analysis, the panel has selected four locations in Indore as possible alternatives, which are location-1 (*F*_1_), location-2 (*F*_2_), location-3 (*F*_3_) and location-4 (*F*_4_). Table S1 presents the sample questionnaire to evaluate the HWRPLs with respect to multiple criteria (see Section S3 in supplementary file). Table 4 and Fig. 2 present the list of criteria obtained from online questionnaire and experts’ opinions. In order to mitigate the subjective randomness, the qualitative data is transformed to FFNs using the tabular values from Tables 2–3. This case study is presented for demonstration purpose of choosing the best HWRPL, which proves the applicability of the developed approach. Readers may diminish or add some attributes as per their requirements.

**Table 4:** Details of criteria for HWRPL assessment^7,61,65^.

Dimensions	Criteria	Meanings
Economic (*L*_1_)	Transportation cost (*V*_1_)	It involves the total costs of transportation used for household waste processing plant location selection
	Recycling cost (*V*_2_)	It contains operation and installation costs of the HWRP
	Construction cost (*V*_3_)	It involves the total amount of costs used in the assessment of HW processing plant location
Social (*L*_2_)	Job creation (*V*_4_)	It considers the formation of new jobs as it produces new service capacity
	Sustainability (*V*_5_)	It considers the recovery and recycling processes conducting in the HWRP
Environmental (*L*_3_)	Amount of household wastes (*V*_6_)	It discusses to household wastes volume to be treated at the processing plant
	Capacity of household waste processing plant (*V*_7_)	It involves the utmost quantity of household wastes that can be treated at the HWRP
	Consumers’ environmental awareness and willingness (*V*_8_)	It considers that whether consumers are willing to release and classify their household wastes to waste collection site
Risk (*L*_4_)	Operational risk (*V*_9_)	It considers that shortage in the quantity of waste transported to processing plant
	Social risk (*V*_10_)	It considers the risk of explosion and fire because of the production of methane gas on processing sites

### Implementation process

Here, we apply the proposed FF-MEREC-SWARA-MARCOS model on the aforesaid case study in order to choose the best HWRPL over multiple criteria. In the following, we provide the computational steps of the developed framework:

**Steps 1–3:** Table 2 presents the QRs and their associated FFNs to state the rating of DMEs and the defined attributes. Tables 3 reports the linguistic values and their corresponding FFNs for evaluating the possible recycling plant locations. Using Table 2 and Eqs. (7a)–(7c), the DMEs’ weights are determined in Table 5. Table 6 presents QDM obtained based on four DMEs (*g*_1_, *g*_2_, *g*_3_, *g*_4_), wherein each QR presents the assessment value of each location *F*_*i*_ against the given attributes. From Eq. (8) and Table 6, an A-FF-DM $M = \left( {\upsilon_{ij} } \right)_{p\, \times \,q}$ is established, given in Table 7.

**Table 5:** Weight of DME for HWRPLs selection.

DEs	QRs	FFNs	Weights
*g*_1_	SS	(0.6,0.7)	0.1252
*g*_2_	ES	(0.9,0.2)	0.3703
*g*_3_	S	(0.7,0.5)	0.2113
*g*_4_	MS	(0.8,0.4)	0.2932

**Table 6:** Qualitative decision matrix constructed by four DMEs.

	*F*_1_	*F*_2_	*F*_3_	*F*_4_
*V*_1_	(QU, SU, SU, U)	(SU, U, A, SU)	(U, SU, A, MU)	(QU, U, SU, SU)
*V*_2_	(A, SU, MU, SU)	(SU, QU, SU, A)	(U, SU, QU, QU)	(MU, QU, SU, QU)
*V*_3_	(U, U, SU, A)	(U, MU, A, QU)	(SU, U, U, SU)	(U, A, U, U)
*V*_4_	(A, SP, QP, P)	(SP, A, MP, P)	(QP, A, MP, SP)	(QP, SP, A, MP)
*V*_5_	(MP, A, A, QP)	(QP, A, QP, EP)	(A, QP, P, A)	(QP, SP, A, A)
*V*_6_	(QP, SP, QP, A)	(MP, SP, SP, A)	(QP, MP, A, SP)	(QP, QP, SP, EP)
*V*_7_	(A, SP, QP, MP)	(SP, QP, P, SP)	(A, MP, QP, EP)	(QP, QP, QP, SP)
*V*_8_	(SP, A, QP, P)	(P, A, P, A)	(QP, SP, QP, SP)	(QP, QP, A, P)
*V*_9_	(QU, A, SU, U)	(MP, SU, A, QU)	(U, SU, SU, SU)	(U, QU, MU, A)
*V*_10_	(SU, MU, SU, SU)	(A, U, U, MU)	(SU, A, QU, QU)	(QU, SU, SU, QU)

**Table 7:** The A-FF-DM for HWRPLs evaluation.

	*F*_1_	*F*_2_	*F*_3_	*F*_4_
*V*_1_	(0.373,0.861)	(0.410,0.867)	(0.410,0.843)	(0.340,0.899)
*V*_2_	(0.363,0.871)	(0.373,0.873)	(0.323,0.878)	(0.333,0.892)
*V*_3_	(0.274,0.917)	(0.379,0.898)	(0.295,0.915)	(0.339,0.846)
*V*_4_	(0.605,0.684)	(0.679,0.637)	(0.689,0.623)	(0.574,0.704)
*V*_5_	(0.601,0.699)	(0.620,0.694)	(0.650,0.610)	(0.574,0.704)
*V*_6_	(0.644,0.671)	(0.665,0.639)	(0.640,0.544)	(0.635,0.637)
*V*_7_	(0.605,0.684)	(0.671,0.598)	(0.664,0.529)	(0.656,0.613)
*V*_8_	(0.605,0.710)	(0.666,0.644)	(0.644,0.655)	(0.591,0.658)
*V*_9_	(0.402,0.819)	(0.406,0.848)	(0.361,0.867)	(0.237,0.919)
*V*_10_	(0.361,0.910)	(0.339,0.899)	(0.390,0.823)	(0.373,0.861)

**Fig. 3:**
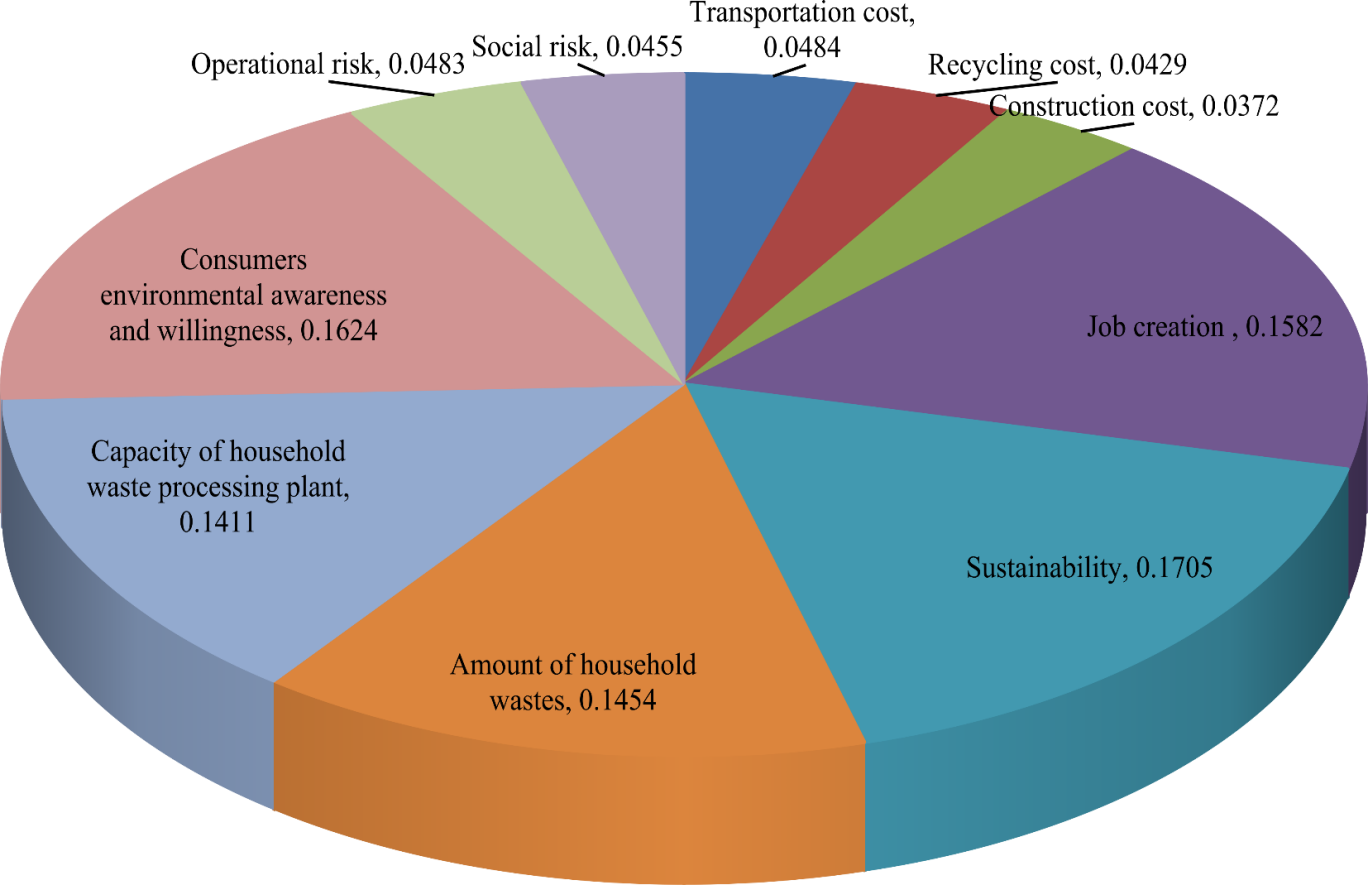
Presentation of objective weight by FF-MEREC for HWRPLs assessment.

**Step 4:** Since some sustainability indicators are of benefit-type and remaining are cost-type, so that we create NA-FF-DM in Table 8 using Eq. (9). Further, we constructed the score matrix based on Eq. (10). To obtain the objective weight with the FF-MEREC model, we find the complete performance of each location using Eq. (11) and given as $\Psi_{1}$ = 0.407, $\Psi_{2}$ = 0.366, $\Psi_{3}$ = 0.353 and $\Psi_{4}$ = 0.392. Then, the overall performance of each location is determined by considering the removal of each criterion using Eq. (12) and presented in Table 9. From Eq. (13), we derive the addition of absolute derivations and finally calculate weight of each criterion through Eq. (14) (see Fig. 3).

**Table 8:** Normalized A-FF-DM for HWRPLs evaluation.

	*F*_1_	*F*_2_	*F*_3_	*F*_4_
*V*_1_	(0.861,0.373)	(0.867,0.410)	(0.843,0.410)	(0.899,0.340)
*V*_2_	(0.871,0.363)	(0.873,0.373)	(0.878,0.323)	(0.892,0.333)
*V*_3_	(0.917,0.274)	(0.898,0.379)	(0.915,0.295)	(0.846,0.339)
*V*_4_	(0.605,0.684)	(0.679,0.637)	(0.689,0.623)	(0.574,0.704)
*V*_5_	(0.601,0.699)	(0.620,0.694)	(0.650,0.610)	(0.574,0.704)
*V*_6_	(0.644,0.671)	(0.665,0.639)	(0.640,0.544)	(0.635,0.637)
*V*_7_	(0.605,0.684)	(0.671,0.598)	(0.664,0.529)	(0.656,0.613)
*V*_8_	(0.605,0.710)	(0.666,0.644)	(0.644,0.655)	(0.591,0.658)
*V*_9_	(0.819,0.402)	(0.848,0.406)	(0.867,0.361)	(0.919,0.237)
*V*_10_	(0.910,0.361)	(0.899,0.339)	(0.823,0.390)	(0.861,0.373)

**Table 9:** Objective weight by FF-MEREC for HWRPLs assessment.

Parameters	$\left( {\Psi_{ij}^{\prime} } \right)$ values				$\mho_{j}$	$\omega_{j}^{o}$
	$\Psi_{1}^{\prime}$	$\Psi_{2}^{\prime}$	$\Psi_{3}^{\prime}$	$\Psi_{4}^{\prime}$		
*V*_1_	0.391	0.350	0.334	0.380	0.062	0.0484
*V*_2_	0.393	0.351	0.339	0.380	0.055	0.0429
*V*_3_	0.398	0.354	0.343	0.375	0.048	0.0372
*V*_4_	0.353	0.321	0.309	0.331	0.204	0.1582
*V*_5_	0.350	0.310	0.307	0.331	0.220	0.1705
*V*_6_	0.357	0.319	0.310	0.344	0.188	0.1454
*V*_7_	0.353	0.323	0.313	0.347	0.182	0.1411
*V*_8_	0.349	0.319	0.302	0.338	0.209	0.1624
*V*_9_	0.387	0.348	0.337	0.383	0.062	0.0483
*V*_10_	0.396	0.355	0.333	0.376	0.059	0.0455

**Fig. 4:**
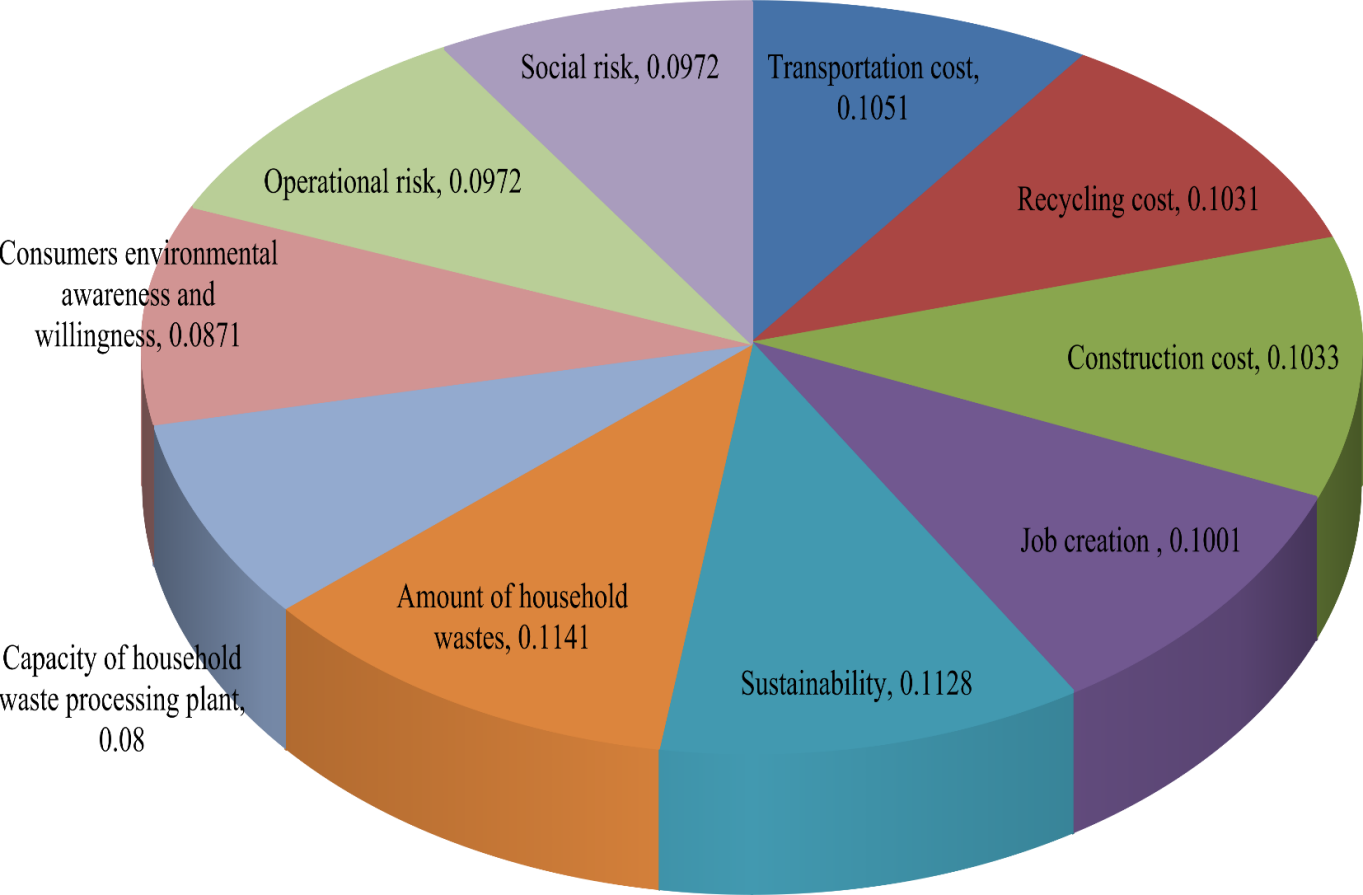
Presentation of subjective weight by the FF-SWARA for locations assessment.

**Fig. 5:**
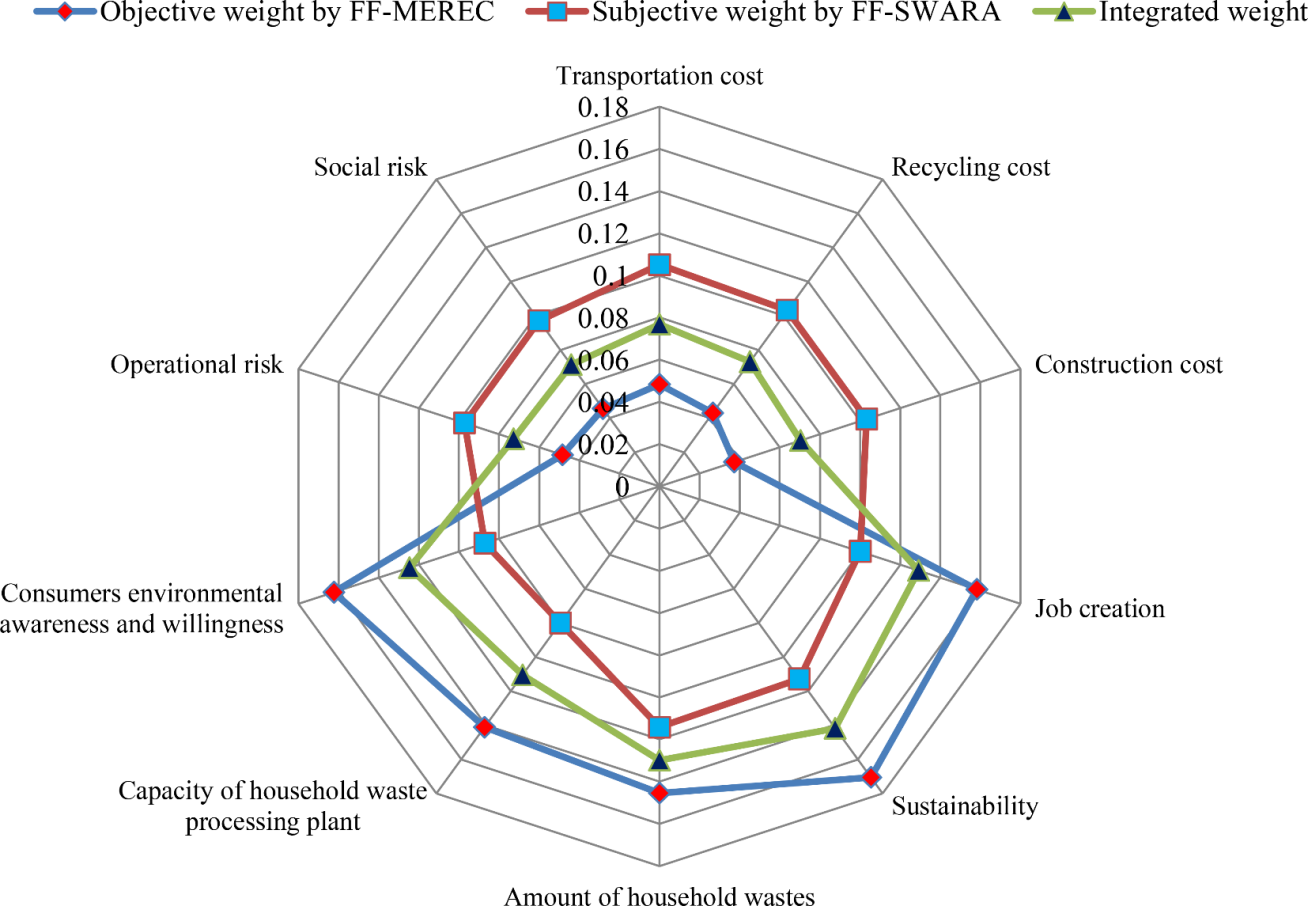
Weight of indicator for locations assessment with the FF-MEREC-SWARA model.

Initially, we calculate the assessment ratings and FF-score values of each criterion provided DMEs from Eq. (5), and given in Table 10. From FF-SWARA model given by Eqs. (15)–(17), we computed the subjective weight with the FF-SWARA model and shown in Table 11 (see Fig. 4). In this context, $\omega_{j}^{s}$ denotes subjective weight of criteria with FF-SWARA and given as $\omega_{j}^{s} =$(0.1051, 0.1031, 0.1033, 0.1001, 0.1128, 0.1141, 0.0800, 0.0871, 0.0972, 0.0972). Next, we have combined the weights obtained by FF-MEREC and FF-SWARA through Eq. (18). Thus, the combined weight set for $\vartheta =0.5$ is graphically shown in Fig. 5 and presented as $\omega_{j} =$ (0.0768, 0.0730, 0.0703, 0.1292, 0.1417, 0.1298, 0.1105, 0.1247, 0.0728, 0.0713).

**Table 10:** The FF-score values of criteria for locations assessment by DMEs.

Criteria	*g*_1_	*g*_2_	*g*_3_	*g*_4_	A-FFNs	Score rating
*V*_1_	A	SP	P	QP	(0.638,0.652)	0.509
*V*_2_	SP	QP	QP	P	(0.644,0.627)	0.490
*V*_3_	SQP	SP	QP	MP	(0.644,0.655)	0.492
*V*_4_	MP	QU	QP	A	(0.651,0.700)	0.467
*V*_5_	A	SP	SP	P	(0.578,0.711)	0.583
*V*_6_	A	A	QP	SP	(0.576,0.724)	0.594
*V*_7_	A	SU	SU	P	(0.417,0.840)	0.240
*V*_8_	QP	A	QU	SU	(0.501,0.776)	0.329
*V*_9_	SP	SU	P	A	(0.629,0.712)	0.444
*V*_10_	P	A	SP	P	(0.615,0.701)	0.444

**Table 11:** Subjective weight using the FF-SWARA approach.

Criteria	Crisp degrees	*s*_*j*_	*k*_*j*_	*r*_*j*_	$\omega_{j}^{s}$
*V*_6_	0.594	-	1.0	1.0	0.1141
*V*_5_	0.583	0.011	1.011	0.9891	0.1128
*V*_1_	0.509	0.074	1.074	0.9209	0.1051
*V*_3_	0.492	0.017	1.017	0.9055	0.1033
*V*_2_	0.490	0.002	1.002	0.9037	0.1031
*V*_4_	0.467	0.023	1.023	0.8774	0.1001
*V*_9_	0.444	0.023	1.023	0.8518	0.0972
*V*_10_	0.444	0.000	1.000	0.8518	0.0972
*V*_8_	0.329	0.115	1.115	0.7639	0.0871
*V*_7_	0.240	0.089	1.089	0.7015	0.0800

Here, Fig. 5 exhibits weight of diverse attributes for HWRPLs assessment. The factor distance to the sustainability (*V*_5_) (0.1417) has been obtained the most essential attributes for HWRPLs assessment. Amount of household wastes (*V*_6_) (0.1298) is the second essential factor for HWRPLs assessment. Job creation (*V*_4_) with weight 0.1292 is the third significant attribute for HWRPLs assessment and remaining attributes are taken as essential attribute for HWRPLs assessment.

**Step 6:** Apply Eqs. (19)–(20) and Table 6, the PIR and NIR on FFNs are presented as follows:

$$\alpha_{j}^{ + } \,$$** = **{(0.34, 0.899), (0.333, 0.892), (0.274, 0.917), (0.689, 0.623), (0.65, 0.61), (0.64, 0.544), (0.664, 0.529), (0.666, 0.644), (0.237, 0.919), (0.361, 0.91)}.

$$\alpha_{j}^{ - } \,$$** = **{(0.41, 0.843), (0.363, 0.871), (0.339, 0.846), (0.574, 0.704), (0.574, 0.704), (0.644, 0.671), (0.605, 0.684), (0.605, 0.71), (0.402, 0.819), (0.39, 0.823)}.

**Step 7:** From Eq. (21) and Table 8, the WNA-FF-DM for HWRPLs assessment is created and is mentioned in Table 12.

**Table 12:** A WNA-FF-DM for HWRPLs assessment.

	*F*_1_	*F*_2_	*F*_3_	*F*_4_	$\alpha_{jw}^{ + }$	$\alpha_{jw}^{ - }$
*V*_1_	(0.422,0.927)	(0.427,0.934)	(0.407,0.934)	(0.456,0.920)	(0.456,0.921)	(0.408,0.934)
*V*_2_	(0.423,0.929)	(0.425,0.930)	(0.429,0.921)	(0.442,0.923)	(0.442,0.923)	(0.423,0.929)
*V*_3_	(0.462,0.913)	(0.442,0.934)	(0.459,0.918)	(0.398,0.927)	(0.462,0.913)	(0.398,0.927)
*V*_4_	(0.317,0.952)	(0.362,0.943)	(0.368,0.941)	(0.299,0.956)	(0.368,0.941)	(0.299,0.956)
*V*_5_	(0.324,0.951)	(0.336,0.950)	(0.354,0.932)	(0.308,0.951)	(0.354,0.932)	(0.308,0.951)
*V*_6_	(0.340,0.949)	(0.353,0.944)	(0.338,0.924)	(0.335,0.943)	(0.338,0.924)	(0.341,0.950)
*V*_7_	(0.301,0.959)	(0.339,0.945)	(0.335,0.932)	(0.330,0.947)	(0.335,0.932)	(0.301,0.959)
*V*_8_	(0.313,0.958)	(0.350,0.947)	(0.336,0.949)	(0.305,0.949)	(0.350,0.947)	(0.313,0.958)
*V*_9_	(0.383,0.936)	(0.405,0.936)	(0.419,0.929)	(0.469,0.901)	(0.469,0.901)	(0.383,0.936)
*V*_10_	(0.457,0.930)	(0.446,0.926)	(0.384,0.935)	(0.412,0.932)	(0.456,0.930)	(0.384,0.935)

**Step 8:** Using Eq. (22) and Table 12, the FF-score ratings of options, weighted PIR and NIR are obtained and given in Table 13.

**Table 13:** FF-score ratings of each option for HWRPLs evaluation.

	*F*_1_	*F*_2_	*F*_3_	*F*_4_	$\alpha_{jw}^{ + }$	$\alpha_{jw}^{ - }$
*V*_1_	0.139	0.132	0.127	0.157	0.157	0.127
*V*_2_	0.138	0.136	0.149	0.150	0.150	0.136
*V*_3_	0.169	0.136	0.162	0.134	0.169	0.134
*V*_4_	0.084	0.104	0.109	0.077	0.109	0.077
*V*_5_	0.088	0.091	0.117	0.084	0.117	0.084
*V*_6_	0.092	0.102	0.125	0.099	0.125	0.092
*V*_7_	0.073	0.098	0.114	0.093	0.114	0.073
*V*_8_	0.076	0.097	0.092	0.087	0.097	0.076
*V*_9_	0.118	0.123	0.136	0.186	0.186	0.118
*V*_10_	0.145	0.148	0.120	0.130	0.148	0.120
$\Delta_{i}$	1.121	1.165	1.25	1.198	1.37	1.036

**Steps 9–11:** Applying Eqs. (23)–(24), we obtain UDs of options as $m_{1}^{ + } \, =$ 0.819, $m_{2}^{ + } \, =$ 0.851, $m_{3}^{ + } \, =$ 0.913, $m_{4}^{ + } \, =$ 0.875, $m_{1}^{ - } \, =$ 1.082, $m_{2}^{ - } \, =$ 1.124, $m_{3}^{ - } \, =$ 1.206, $m_{4}^{ - } \, =$ 1.156 and the CUFs of options as $g\left( {m_{1} } \right)\, =$ 0.617, $g\left( {m_{2} } \right)\, =$ 0.642, $g\left( {m_{3} } \right)\, =$ 0.688 and $g\left( {m_{4} } \right)\, =$ 0.66. Thus, prioritization of HWRPL options is $F_{3} \succ F_{4} \succ F_{2} \succ F_{1}$ and the HWRPL-3 (*F*_3_) is best choice with the maximum CUF.

### Comparative study

In the following, we present comparison of developed and extant MCDM approaches under the context of FFSs. In this regard, some MCDM methods are chosen, which are Mishra & Rani’s WASPAS model^66^, Gül’s ARAS model^67^, Simić et al.’s CoCoSo model^68^ and Senapati & Yager’s TOPSIS model^20^ approaches are employed to deal aforesaid problem.

#### Mishra & Rani’s WASPAS model

**Steps 1–4:** Follow the steps of developed framework.

**Step 5:** Finding the values of weighted sum rating and product rating using Eq. (25) and Eq. (26), respectively.

25 $$\wp_{i}^{(1)} \, = \,\mathop \oplus \limits_{j = 1}^{q} \omega_{j} \,\varsigma_{ij} ,$$

26 $$\wp_{i}^{(2)} \, = \,\mathop \otimes \limits_{j = 1}^{q} \varsigma_{ij}^{{\omega_{j} }} ,\,i = 1,2,...,p.$$

**Step 6:** Estimation of UD of options as

27 $$Q_{i} \, = \,\iota \,\wp_{i}^{(1)} \, + \,\left( {1 - \,\iota } \right)\,\wp_{i}^{(2)} ,\,i = 1,2,...,p.$$

where $\iota$ is the utility coefficient within the range of 0 to 1.

**Step 7:** Choosing the best option with highest FF-score rating of UDs.

From Eqs. (25) to (27), the UD of option for HWRPLs are demonstrated in Table 14.

**Table 14:** UDs of options for locations assessment.

Options	$\wp_{i}^{(1)} \,$	$\wp_{i}^{(2)} \,$	${\mathbb{S}}\left( {\wp_{i}^{(1)} \,} \right)$	${\mathbb{S}}\left( {\wp_{i}^{(2)} } \right)\,$	$Q_{i}$	Ranks
*F*_1_	(0.762,0.54)	(0.697,0.619)	0.643	0.551	0.6427	4
*F*_2_	(0.776,0.532)	(0.731,0.584)	0.658	0.596	0.6582	3
*F*_3_	(0.768,0.491,0.754)	(0.726,0.540,0.772)	0.667	0.612	0.6670	1
*F*_4_	(0.766,0.509,0.748)	(0.693,0.597,0.769)	0.659	0.560	0.6588	2

Therefore, the ranking of option for locations assessment is $F_{3} \succ F_{4} \succ F_{2} \succ F_{1}$ and the option location-3 (*F*_3_) is an ideal location with maximum degree.

#### Gül’s ARAS model

**Steps 1–4:** Similar to the developed framework.

**Step 5:** Defining an “optimal alternative rating (OAR)”.

28 $$h_{0} = \left( {\hbar_{j}^{0} ,\,{{\lambda}}_{j}^{0} } \right) = \,\left\{ \begin{gathered} \left( {\mathop {\max }\limits_{i} \,\hbar_{ij} ,\mathop {\min }\limits_{i} \,{{\lambda}}_{ij} } \right),\,\,\,j\, \in \,V_{b} ,\,\, \hfill \\ \left( {\mathop {\min }\limits_{i} \,\hbar_{ij} \mathop {\max }\limits_{i} \,{{\lambda}}_{ij} } \right),\,\,\,j\, \in \,V_{n} . \hfill \\ \end{gathered} \right.\,\,$$

**Step 6:** Find the “relative assessment rating (RAR)” and the UD of each option.

Using the WNA-FF-DM $${\mathbb{N}}_{w} = \left( {\overset{\lower0.5em\hbox{$\smash{\scriptscriptstyle\frown}$}}{\varsigma }_{ij} } \right)_{p\, \times \,q}$$, given in Eq. (21), the RAR of option is obtained as

29 $$\Delta_{i} \, = \,\sum\limits_{j = 1}^{q} {{\mathbb{S}}\left( {\overset{\lower0.5em\hbox{$\smash{\scriptscriptstyle\frown}$}}{\varsigma }_{ij} } \right)} ,\,i = 1,2,...,p.$$

The UD is computed using the RAR $\left({\Delta }_{i}\right)$ and OAR $\left({h}_{0}\right)$. The UD $\left({Q}_{i}\right)$ of option ${F}_{i},\, i=\text{1,2}, \dots ,p$ is given as

30 $$Q_{i} = \frac{{\Delta_{i}}}{{h_{0} }},i = 1,2,...,p.$$

The prioritize the option in ascending UD $\left({Q}_{i}\right)$, $i=\text{1,2}, \dots ,p$.

From Eq. (28) and the A-FF-DM, we define the OAR to the HWRPLs assessment as follows:

$h_{0} \,$** = {**(0.34, 0.899), (0.333, 0.892), (0.274, 0.917), (0.689, 0.623), (0.65, 0.61), (0.64, 0.544), (0.664, 0.529), (0.666, 0.644), (0.237, 0.919), (0.361, 0.91)**}.** Using Eq. (5) and Eq. (29), we obtain the ROR of HWRPLs assessment. Applying Eq. (30), the UD *Q*_*i*_ is computed as *Q*_1_ = 0.8187, *Q*_2_ = 0.8506, *Q*_3_ = 0.9128 and *Q*_4_ = 0.8746. Based on the UD (*Q*_*i*_), the prioritization of sites to establish new HWRP is $F_{3} \succ F_{4} \succ F_{2} \succ F_{1}$ and thus, the location-3 (*F*_3_) is the ideal location over various criteria.

#### *Simic *et al*.’s FF-CoCoSo model*

**Steps 1–5:** Follow to FF-WASPAS framework.

**Step 6:** Estimating the “relative degree (RD)” of each option as

31 $$t_{i}^{\left( 1 \right)} = \frac{{{\mathbb{S}}\left( {\wp_{i}^{(1)} } \right) + {\mathbb{S}}\left( {\wp_{i}^{(2)} } \right)}}{{\sum\limits_{i = 1}^{p} {\left( {{\mathbb{S}}\left( {\wp_{i}^{(1)} } \right) + {\mathbb{S}}\left( {\wp_{i}^{(2)} } \right)} \right)} }},$$

32 $$t_{i}^{\left( 2 \right)} = \frac{{{\mathbb{S}}\left( {\wp_{i}^{(1)} } \right)}}{{\mathop {\min }\limits_{i} {\mathbb{S}}\left( {\wp_{i}^{(1)} } \right)}} + \frac{{{\mathbb{S}}\left( {\wp_{i}^{(2)} } \right)}}{{\mathop {\min }\limits_{i} {\mathbb{S}}\left( {\wp_{i}^{(2)} } \right)}},$$

33 $$t_{i}^{\left( 3 \right)} = \frac{{\vartheta \,{\mathbb{S}}\left( {\wp_{i}^{(1)} } \right) + \left( {1 - \vartheta } \right){\mathbb{S}}\left( {\wp_{i}^{(2)} } \right)}}{{\mathop {\vartheta \,\max }\limits_{i} {\mathbb{S}}\left( {\wp_{i}^{(1)} } \right) + \left( {1 - \vartheta } \right)\mathop {\max }\limits_{i} {\mathbb{S}}\left( {\wp_{i}^{(2)} } \right)}},i = 1,2,...,p,$$

where *ϑ* is the RCR coefficient within the range of 0 to 1.

**Step 7:** Assessment of the compromise rating (CR) of options.

The CR $\left( {t_{i} } \right)$ of each option is given by

34 $$t_{i} = \left( {t_{i}^{\left( 1 \right)} t_{i}^{\left( 2 \right)} t_{i}^{\left( 3 \right)} } \right)^{{{\raise0.7ex\hbox{$1$} \!\mathord{\left/ {\vphantom {1 3}}\right.\kern-0pt} \!\lower0.7ex\hbox{$3$}}}} + \frac{1}{3}\left( {t_{i}^{\left( 1 \right)} + t_{i}^{\left( 2 \right)} + t_{i}^{\left( 3 \right)} } \right),\,i = 1,2,...,p.$$

Hence, choose the best option with the highest CD $\left( {t_{i} } \right).$

Apply Eqs. (31)–(33), the RDs of HWRPLs assessment is presented in Table 15. From Eq. (34), the CR of each HWRPL is calculated and is mentioned in Table 15. As per CRs, the preference of HWRPLs is $F_{3} \succ F_{4} \succ F_{2} \succ F_{1} ,$ and thus, the HWRPL-3 (*F*_3_) is the best site over different criteria.

**Table 15:** The RDs and CRs findings for HWRPLs assessment.

	$t_{i}^{\left( 1 \right)}$	$t_{i}^{\left( 2 \right)}$	$t_{i}^{\left( 3 \right)}$	$t_{i}$	Ranking
*F*_1_	0.2413	2.0	0.9635	1.843	4
*F*_2_	0.2535	2.106	0.9867	1.923	2
*F*_3_	0.2587	2.15	1.0	1.959	1
*F*_4_	0.2465	2.042	0.9877	1.8845	3

#### FF-TOPSIS method

The procedure for FF-TOPSIS is.

**Steps 1–5:** Similar to the proposed approach.

**Step 6:** Computing the weighted FF-distances $E_{i}^{ + } = \,dis\left( {\upsilon_{ij},\,\alpha^{ + } } \right)$ and $E_{i}^{ - } = dis\left( {\upsilon_{ij},\,\alpha^{ - } } \right)$ of options based on the FF-Euclidean distance measure^20^.

**Step 7:** The relative closeness rating (RCR) of options from FF-PIR is estimated in the given expression:

35 $$R\left( {F_{i} } \right) = \frac{{E_{i}^{ - } }}{{E_{i}^{ + } + \,E_{i}^{ - } }},\,\,\forall \,i.$$

Rank the options according to the RCRs.

**Fig. 6:**
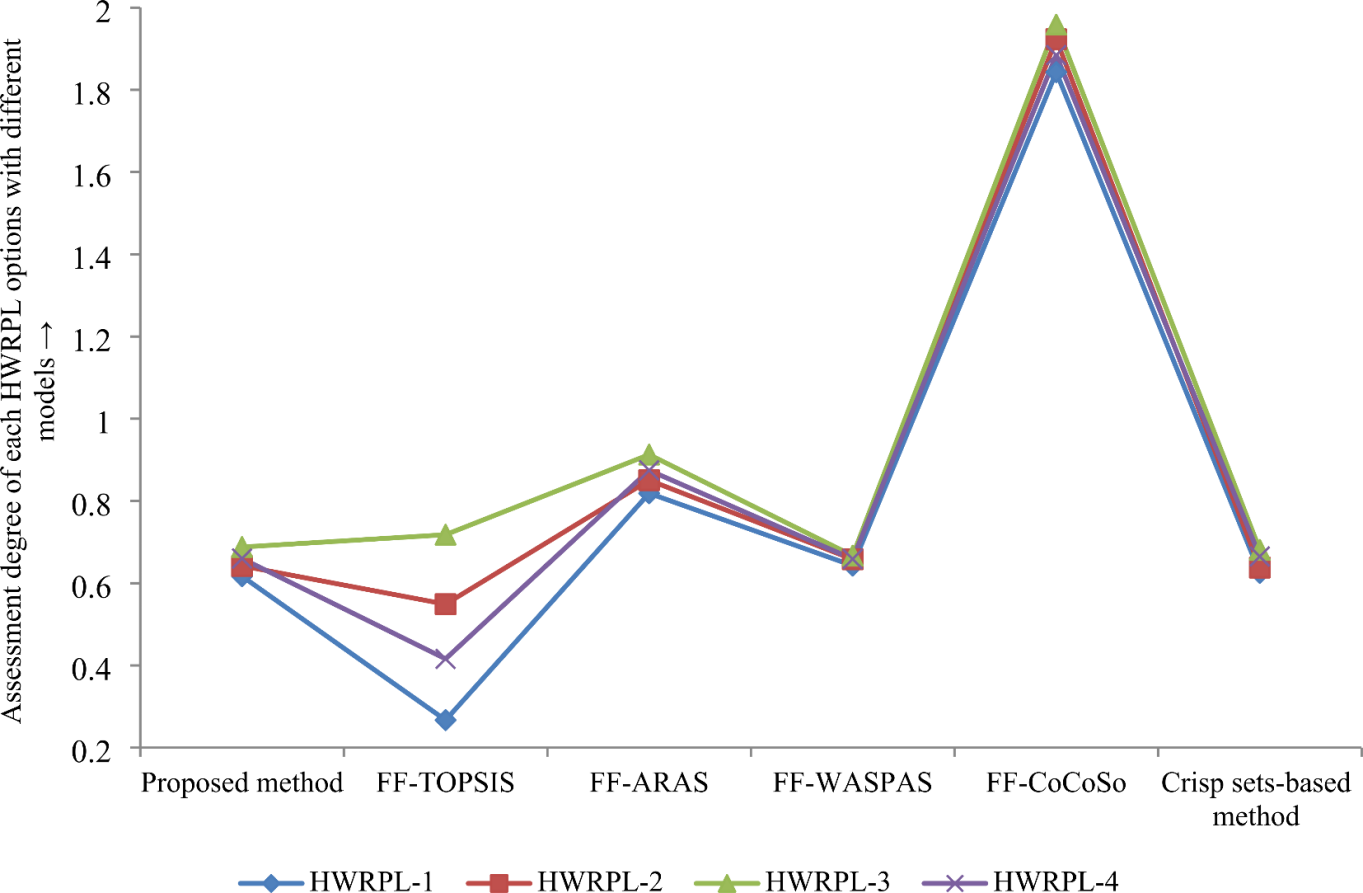
Ranking order for HWRPL assessment with different methods.

We have implemented the TOPSIS method on the aforesaid case study. We have computed FF-distance measures as follows: $E_{1}^{ + } =$ 0.096, $E_{1}^{ - } =$ 0.035, $E_{2}^{ + } =$ 0.062, $E_{2}^{ - } =$ 0.076, $E_{3}^{ + } =$ 0.037, $E_{3}^{ - } =$ 0.094, $E_{4}^{ + } =$ 0.082 and $E_{4}^{ - } =$ 0.058. Based on Eq. (35), the relative closeness coefficient to the FF-PIR is presented as follows: $R\left( {F_{1} } \right)$ = 0.267, $R\left( {F_{2} } \right)$ = 0.549, $R\left( {F_{3} } \right)$ = 0.718, and $R\left( {F_{4} } \right)$ = 0.416. The ranking of plant locations is $F_{3} \succ F_{2} \succ F_{4} \succ F_{1} ,$ thus, the location-3 (*F*_3_) is the best location for establishing the HWRP. The key benefits of the proposed hybrid framework are presented as follows (see Fig. 6):

- The score values used by^20,55,56^ has counter intuitive problem in some cases, while the developed score function avoids these limitations. Thus, the developed FF-score function can successfully offer the ranks of the FFNs.
- The FF-distance measure developed in this paper evades the drawbacks of various extant FF-distance measures by^57–59^. Further, the DMEs’ weights are computed through the proposed distance FF-distance measure in the presented FF-MEREC-SWARA-MARCOS method. Thus, the proposed model provides more accurate result than existing models.
- The proposed FF-MEREC-SWARA-MARCOS framework estimates the weight of attributes using FF-MEREC-SWARA approach integrating the objective and subjective weights of attributes, which achieves the weight values of attributes from most favourable ways, whereas in the Mishra and Rani’s WASPAS approach, objective weight of attribute is obtained with FF-similarity measure and FF-score rating-based approach, in the Simic et al.’s CoCoSo approach, only objective weight of attribute is taken with similarity measure, in the FF-ARAS, only objective weight of attribute is estimated by MEREC tool and in the FF-TOPSIS model, attribute weight is taken arbitrarily.
- Comparing with diverse extant models, we find suitable HWRPL-3 (*F*_3_) is the same as the developed hybrid framework. The CUFs of the proposed FF-MEREC-SWARA-MARCOS approach have been estimated with FF-PIR and FF-NIR, while Mishra and Rani’s WASPAS and Simic et al.’s CoCoSo models employ averaging and geometric AOs and the FF-ARAS utilizes averaging AO with FF-PIR to obtain final rating of options. Hence, proposed FF-MEREC-SWARA-MARCOS approach is more comprehensive and more flexible. Considering this feature, the proposed FF-MEREC-SWARA-MARCOS approach can be applied more broadly.

### Sensitivity investigation

**Fig. 7:**
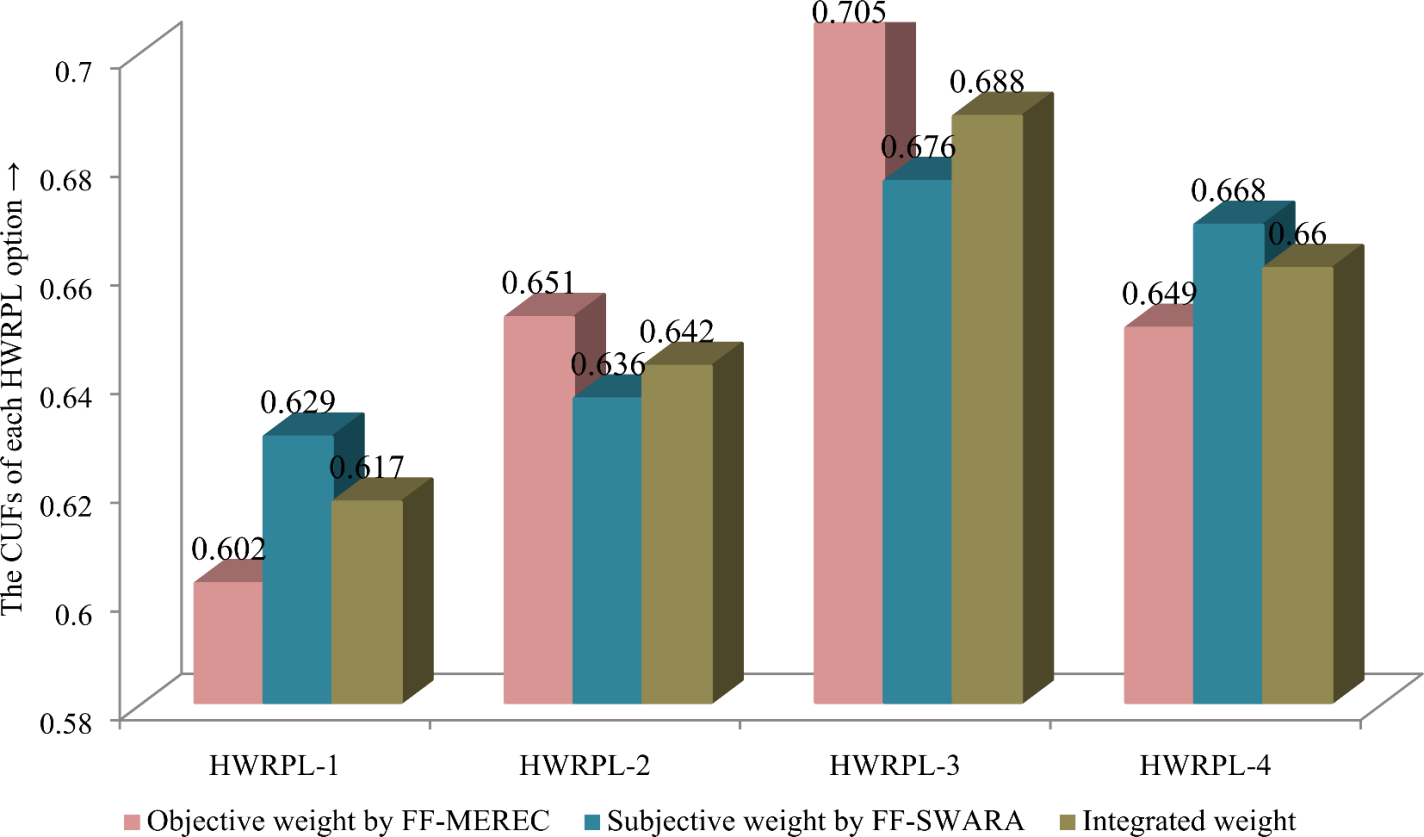
Changes in CUF ratings for locations assessment with different models.

We study changes in CUF ratings and preferences of HWRPLs over changing the weights of diverse attributes from objective and subjective weights using “FF-MEREC-SWARA” model for HWRPLs assessment. The prioritizations of locations of HWRP evaluations are obtained over the objective, the combined and the subjective weights of factors using the FF-MEREC-SWARA models and are discussed in Table 16 and Fig. 7. Apply the FF-MEREC approach, the CUF ratings and preferences of HWRPLs are estimated as *F*_1_ = 0*.*602, *F*_2_ = 0*.*651, *F*_3_ = 0*.*705 and *F*_4_ = 0*.*649 and prioritization of locations is given as $F_{3} \succ F_{2} \succ F_{4} \succ F_{1} .$ Utilizing the FF-SWARA model, the CUF ratings and preferences of HWRPLs are obtained as *F*_1_ = 0*.*629, *F*_2_ = 0*.*636, *F*_3_ = 0*.*676 and *F*_4_ = 0*.*668 and the ranking order of HWRPLs is given as $F_{3} \succ F_{4} \succ F_{2} \succ F_{1} .$ Thus, we conclude that the suitable HWRPL choice considering all types of weight evaluating approach is the same, i.e., HWRPL-3 (*F*_3_). Hence, as per aforementioned study, it is found that the positioning of different significant degree of strategy parameter (*ϑ*) will improve the performance of proposed FF-MEREC-SWARA-MARCOS methodology.

**Table 16:** The CUFs for locations assessment with diverse models.

Approaches	CUFs for HWRPL assessment				Ranks
	*F*_1_	*F*_2_	*F*_3_	*F*_4_	
FF-MEREC for objective weighting	0.602	0.651	0.705	0.649	*F*_3_ $\succ$* F*_2_ $\succ$* F*_4_ $\succ$* F*_1_
FF-SWARA for subjective weighting	0.629	0.636	0.676	0.668	*F*_3_ $\succ$* F*_4_ $\succ$* F*_1_ $\succ$* F*_1_
Integrated method	0.617	0.642	0.688	0.66	*F*_3_ $\succ$* F*_4_ $\succ$* F*_1_ $\succ$* F*_1_

### Implications of the proposed work

This work introduces an innovative approach to select the most suitable site for HWRP construction that approves to enhance the sustainability pillars on FFSs settings. The developed methodology is based on the FF-distance measure, the FF-MEREC, the FF-SWARA and the FF-MARCOS methods called the “FF-MEREC-SWARA-MARCOS” with Fermatean fuzzy information. A case study of HWRPLs selection is taken to validate the results and reasonableness of developed framework. This methodology not only prioritizes the locations with diverse sustainability perspectives, but also recognizes the significance values of DMEs using novel formula and the criteria using integrated weighting tool. Furthermore, this paper discusses new FF-distance measure and FF-score function for FFSs and verifies their usefulness over the formerly proposed FF-distance measures and FF-score/accuracy functions.

Moreover, the comparative assessment with extant procedures namely, the FF-TOPSIS, the FF-ARAS, the FF-WASPAS and the FF-CoCoSo has also shown to elucidate the reasonableness of proposed approach. The findings show that HWRPL-3 (*F*_3_) is the best choice for constructing the HWRP, whereas the preferences of HWRPLs, determined with the proposed approach and extant models, are slightly vary. We observe that the variation in the prioritizations of HWRPLs is owing to the following causes. The proposed approach provides significance to DMEs’ preferences in the assessment of alternatives and factors. Based on the introduced method, group of DMEs focuses not only in the beneficial factors but also studies the non-beneficial factors. Additionally, sensitivity investigation over different ratings of coefficient ‘*ϑ*’ has implemented to validate the permanence of proposed approach and thus, we form that HWRPL-3 (*F*_3_) is the optimal choice for HWRP establishment.

This work recommends stakeholders/representatives to realize the performance of HWRPL options with diverse features of sustainability under uncertainty setting. The proposed FF-MEREC-SWARA-MARCOS framework has subsequent implications for experts and researchers:

- The most optimal HWRPL candidate executes better over economic, social, environmental and risk aspects of sustainability with least cost and positive impacts on environment.
- Executives and stakeholders can utilize data discussed in this work to assist their decision for locating appropriate HWRPs.
- The proposed FF-MEREC-SWARA-MARCOS approach not only appraises the importance ratings of aforesaid factors but also deals vagueness and fuzziness obtained in the procedure of HWRPLs evaluation.

## Conclusions

The current study aims to propose hybrid MCDM framework for assessing recycling plant locations for household waste from sustainable perspectives. For this purpose, based on literature review and experts’ knowledge, 10 criteria were selected from sustainability perspectives including economic, social, environmental and risk dimensions. This methodological framework has been incorporated the proposed FF-distance measure-based model, proposed FF-score function, the FF-MEREC model, the FF-SWARA model and the FF-MARCOS method from Fermatean fuzzy scenario called the “FF-MEREC-SWARA-MARCOS” model. In this context, to rank the FFNs, new FF-score function has been introduced, which evades the drawbacks of extant FF-score functions^20,55,56^. Also, to quantify the distance between FFSs, new FF-distance measure has been proposed with some elegant axioms. The developed FF-distance measures can deal the weaknesses of extant FF-distance measures Ashraf et al.’s^57^ distance measures, Ganie’s^58^ distance measures and Kirisci’s^59^ distance measure) between FFSs. Further, developed hybrid framework has been used on a case study of household waste recycling plant location (HWRPL) selection problem, which confirms the applicability and efficacy. Comparison and sensitivity investigation have been made to reveal validity of obtained outcomes. Based on the comparison with extant approaches, we have found that the presented model is simple, easy-to-use and reliable in order to tackle realistic decision-making problems. This work makes an innovative contribution in the form of a hybrid FF-MEREC-SWARA-MARCOS decision support system, which can be used by the household waste recycling plant construction companies for the selection of location sustainable from sustainability perspective. Furthermore, the proposed hybrid system makes realistic contributions for outlining policies and strategies for solid waste management activities and household waste recycling plant construction. The limitation of the developed approach is as (1) all criteria are assumed to be independent. In effect, there are interrelationships among criteria in realistic group decision-making problems, and (2) the assessment ranking procedures should include more decision experts to provide more concise and valid outcomes.

In future, we will consider the technological aspects of criteria and stakeholders’ preferences during the evaluation of household waste recycling plant sites. Furthermore, we will extend developed approach on diverse fuzzy settings such as “Fermatean rough fuzzy sets (FRFSs)”, “interval-valued hesitant rough fuzzy sets (IVHRFSs)” and “complex fuzzy sets (CFSs)” to tackle more vagueness of DMEs’ subjective decisions.

## Electronic supplementary material

Below is the link to the electronic supplementary material.

Supplementary Material 1

## Data Availability

All data generated or analyzed during this study are included in this published article.
