# Supplementary material for: Fermatean fuzzy score function and distance measure based group decision making framework for household waste recycling plant location selection
Source: Sci Rep. 2024 Nov 15;14:28106. doi: 10.1038/s41598-024-78158-z (PMC11567974; doi:10.1038/s41598-024-78158-z)
Supplement: Supplementary file 1 — Supplementary Material 1 [file 41598_2024_78158_MOESM1_ESM.docx]

**SUPPLEMENTARY FILE**

**S1. Proofs of Theorems**

**Proof of Theorem 3.1:** Differentiating Eq. (5) partially with respect to , we have

Similarly, Eq. (5) partially with respect to we have

Hence the proof.

**Proof of Theorem 3.3:** (a_1_). If *S* and *T* are two FFSs, then and Thus, it is obvious that $d\left(S, T\right) \geq0.$ On the other hand, $d\left(S, T\right)$ is defined with its normalization, this implies that $d\left(S, T\right) \leq1.$ Hence, $0 \leq d\left(S, T\right) \leq1.$

(a_2_)-(a_3_). These properties are obvious, so we have omitted their proofs.

(a_4_). For given three FFSs *R*, *S* and *T*, if $R \subseteq S\subseteq T,$ then we have and Furthermore, if and then Thus, it follows that

Similarly,

In addition, Therefore, we have

and $\forall e_{i} \in\Omega.$

It implies that $d\left(R, T\right) \geq d\left(R, S\right)$ and $d\left(R, T\right) \geq d\left(S, T\right),$ $\forall R, S, T \in FFSs\left(\Omega\right).$ As Eq. (6) holds all the necessary conditions of distance measure, therefore, it is a valid Fermatean fuzzy distance measure.

**S3. Details of Existing FF-distance measures**

**Ashraf et al. (2023) DMs:**

**Ganie (2022) DMs:**

**Kirisci (2023) DMs:**

$$d_{k}\left(S,T\right)=\frac{1-S_{c}\left(S,T\right)+d_{E}\left(S,T\right)}{2},$$

where

**S3. Sample Questionnaire**

Table S1: Sample questionnaire for HWRP location alternatives evaluation and selection

| Questions | Qualitative rating based on Likert scales | | | | | | | | |  |
| --- | --- | --- | --- | --- | --- | --- | --- | --- | --- | --- |
|  | EP | MP | P | QP | SP | A | SU | QU | U | MU |
| What is your opinion on *LOCATION-1* with respect to *transportation cost*? |  |  |  |  |  |  |  |  |  |  |
| What is your opinion on *LOCATION-1* with respect to *recycling cost*? |  |  |  |  |  |  |  |  |  |  |
| What is your opinion on *LOCATION-1* with respect to *construction cost*? |  |  |  |  |  |  |  |  |  |  |
| What is your opinion on *LOCATION-1* with respect to *job creation*? |  |  |  |  |  |  |  |  |  |  |
| What is your opinion on *LOCATION-1* with respect to *sustainability*? |  |  |  |  |  |  |  |  |  |  |
| What is your opinion on *LOCATION-1* with respect to *amount of household wastes*? |  |  |  |  |  |  |  |  |  |  |
| What is your opinion on *LOCATION-1* with respect to *capacity of household waste processing plant*? |  |  |  |  |  |  |  |  |  |  |
| What is your opinion on *LOCATION-1* with respect to *consumers’ environmental awareness and willingness*? |  |  |  |  |  |  |  |  |  |  |
| What is your opinion on *LOCATION-1* with respect to *operational risk*? |  |  |  |  |  |  |  |  |  |  |
| What is your opinion on *LOCATION-1* with respect to *social risk*? |  |  |  |  |  |  |  |  |  |  |

**S4. Pseudo Code of Proposed Framework**

| **Algorithm:** Pseudo code representation of the proposed FF-MEREC-SWARA-MARCOS model |
| --- |
| **Input:** $p, q\mathcal{, l,}$ where *p*, *q* and $\mathcal{l}$ are the numbers of options, factors and DMEs. |
| **Output:** Rank the household waste recycling power locations (HWRPLs). |
| **Begin** |
| **Step 1:** Input QDM and weight of DME into QTs and covert into FFNs. **# Convert using Tables 3-4**. |
|  |
| **Step 2: For *k* = 1 to** $\mathcal{l}$ Obtaining the FF-score ratings and FF-distance measure values from Eq. (7a) and Eq. (7b).  Compute the weight $\left( \boldsymbol{\Phi}_{\boldsymbol{k}} \right)$ of DME using proposed FF-score function and FF-DM-based approach from Eq. (7c).  **End for** |
| **Step 3: For *i* = 1 to *p***  **For *j* =1 to *q***  Use the FFWA (or FFWG) to output A-FF-DM ***A*** using Eq. (8).  **End for**  **End for** |
| **Step 4: For *j* =1 to *q***  Use Eq. (14) to output the objective weight ($\boldsymbol{\omega}_{\boldsymbol{j}}^{\boldsymbol{0}}$) of attribute.  Use Eq. (17) to output the subjective weight ($\boldsymbol{\omega}_{\boldsymbol{j}}^{\boldsymbol{s}}$) of attribute.  Compute the integrated weight ($\boldsymbol{\omega}_{\boldsymbol{j}}$) of attribute from Eq. (18).  **End for** |
| **Step 5: For *j* = 1 to *q***  **For *i* = 1 to *p***  Calculate the PIR ($\boldsymbol{\alpha}_{\boldsymbol{j}}^{\boldsymbol{+}}$) and NIR ($\boldsymbol{\alpha}_{\boldsymbol{j}}^{\boldsymbol{-}}$) ratings using Eq. (19) and Eq. (20).  **End for**  **End for**  **Step 6: For *i* = 1 to *p***  **For *j* = 1 to *q***  Determining the weighted WNA-FF-DM (WNA-FF-DM) ($\mathbb{N}_{w}$) from Eq. (21).  **End for**  **End for** |
| **Step 7: For *i* = 1 to *p***  Using proposed FF-score function to output the FF-score value ($\boldsymbol{\Delta}_{\boldsymbol{i}}$) of WNA-FF-DM from Eq. (22).  **End for**  **Step 8: For *i* = 1 to *p***  Using PIR ($\boldsymbol{\alpha}_{\boldsymbol{j}}^{\boldsymbol{+}}$) and NIR ($\boldsymbol{\alpha}_{\boldsymbol{j}}^{\boldsymbol{-}}$) ratings to output the utility degrees (UDs) ($\boldsymbol{m}_{\boldsymbol{i}}^{\boldsymbol{+}}$ and $\boldsymbol{m}_{\boldsymbol{i}}^{\boldsymbol{-}}$) of *i*^th^ option from Eq. (23).  **End for** |
| **Step 9: For *i* = 1 to *p***  Using UDs to output combined utility function (CUF) of each option using Eq. (24).  **End for**  **Step 10:** Rank the household waste recycling power locations (HWRPLs) in the decreasing values of CUF. |
|  |
|  |
| **End** |
